# Inhibitors of *O*-Acetylserine Sulfhydrylase with a Cyclopropane-Carboxylic Acid Scaffold Are Effective Colistin Adjuvants in Gram Negative Bacteria

**DOI:** 10.3390/ph15060766

**Published:** 2022-06-20

**Authors:** Giannamaria Annunziato, Costanza Spadini, Marialaura Marchetti, Nina Franko, Marialaura Pavone, Mattia Iannarelli, Agostino Bruno, Marco Pieroni, Stefano Bettati, Clotilde Silvia Cabassi, Barbara Campanini, Gabriele Costantino

**Affiliations:** 1 P4T Group, Food and Drug Department, University of Parma, 43124 Parma, Italy; marialaura.pavone@unipr.it (M.P.); agostino.bruno@unipr.it (A.B.); marco.pieroni@unipr.it (M.P.); gabriele.costantino@unipr.it (G.C.); 2 Operative Unit of Animals Infectious Diseases, Department of Veterinary Science, University of Parma, 43126 Parma, Italy; costanza.spadini@unipr.it (C.S.); mattia.iannarelli@studenti.unipr.it (M.I.); clotildesilvia.cabassi@unipr.it (C.S.C.); 3 Department of Medicine and Surgery, University of Parma, 43126 Parma, Italy; marialaura.marchetti@unipr.it (M.M.); stefano.bettati@unipr.it (S.B.); 4 Laboratory of Biochemistry and Molecular Biology, Food and Drug Department, University of Parma, 43124 Parma, Italy; nina.franko@unipr.it (N.F.); barbara.campanini@unipr.it (B.C.)

**Keywords:** antimicrobial resistance (AMR), drug discovery, OASS inhibitors, antimicrobial adjuvants, colistin

## Abstract

Antibacterial adjuvants are of great significance, since they allow one to downscale the therapeutic dose of conventional antibiotics and reduce the insurgence of antibacterial resistance. Herein, we report that *O*-acetylserine sulfhydrylase (OASS) inhibitors could be used as colistin adjuvants to treat infections caused by critical pathogens spreading worldwide, *Escherichia coli*, *Salmonella enterica* serovar Typhimurium, and *Klebsiella pneumoniae*. Starting from a hit compound endowed with a nanomolar dissociation constant, we have rationally designed and synthesized a series of derivatives to be tested against *S.* Typhimurium OASS isoenzymes, StOASS-A and StOASS-B. All acidic derivatives have shown good activities in the nanomolar range against both OASS isoforms in vitro. Minimal Inhibitory Concentrations (MICs) were then evaluated, as well as compounds’ toxicity. The compounds endowed with good activity in vitro and low cytotoxicity have been challenged as a potential colistin adjuvant against pathogenic bacteria in vitro and the fractional inhibitory concentration (FIC) index has been calculated to define additive or synergistic effects. Finally, the target engagement inside the *S.* Typhimurium cells was confirmed by using a mutant strain in which the OASS enzymes were inactivated. Our results provide a robust proof of principle supporting OASS as a potential nonessential antibacterial target to develop a new class of adjuvants.

## 1. Introduction

Antimicrobial resistance (AMR) is the capability of bacteria to counteract the action of antimicrobial drugs that, consequently, become ineffective. Despite all the efforts pursued during the last decades to handle AMR, today it is still considered a critical threat to global health [1,2,3]. The misuse and overuse of antibiotics have led to the development and to the spreading of multidrug-resistant (MDR) and extensively drug-resistant (XDR) bacteria and a detailed list of resistant microorganisms was published by the World Health Organization (WHO) on the fact sheet of 27 February 2017 [4,5]. These bacteria are resistant to many antibiotic drugs currently used in therapy and their infections are associated with severe illness, high hospitalization costs and mortality [6,7,8,9]. In addition, the absence of new antimicrobial drugs in the pharmaceutical pipeline is further worsening the whole picture [10,11]. Efforts are being made to investigate the potential of unexploited targets and to develop new molecules able to interfere with them, as well as to study the biochemical processes linked to these targets [12,13,14,15]. Promising success has been achieved with those approaches that are focused on small molecules inhibiting non-essential pathways, to minimize the future insurgence of resistance. These molecules are most likely to be used as adjuvants in combination with an antimicrobial drug. Antibiotic adjuvants are compounds that do not kill bacteria directly, but rather enhance the effect of antibiotics, for example, through the inhibition of resistance mechanisms [16,17]. The identification of non-bactericidal adjuvant compounds holds several advantages over the development of new antibiotics, the most significant being the decreased evolutionary pressure on bacteria to evolve resistance to a compound that does not exert bactericidal or growth-inhibitory effects. Indeed, it is known that optimally designed combination therapies have the potential to slow resistance evolution. Moreover, while novel antibiotic targets are limited given both the finite number of essential genes and the extensive exploration that this approach has already received, the adjuvant approach is supposed to take advantage of undiscovered targets and consequently, of the developing of unidentified chemical scaffolds that are able to interact with them [18].

Among the many possible non-essential targets in antibacterial therapy, our research group has focused the attention on the cysteine biosynthetic pathway, the so-called Reductive Sulfur Assimilation Pathway (RSAP) [19]. Cysteine is the precursor of all sulfur-containing biomolecules, including methionine, coenzyme A, biotin, Fe−S clusters, penicillin, and glutathione, that are important for most living organisms. Mammals lack the biosynthetic pathway that leads to the cysteine biosynthesis, while bacteria and plants possess an highly conserved machinery leading to the formation of cysteine [20]. In enteric bacteria, the last two steps of cysteine biosynthesis are catalyzed by two enzymes: serine acetyltransferase (SAT) and *O*-acetylserine sulfhydrylase (OASS). These two enzymes form the “cysteine synthase complex” that is stabilized by the interaction of the C-terminal portion of SAT with the substrate binding pocket of OASS [21]. It is shown that many organisms possess two isoforms of OASS, endowed with high homology: *O*-acetylserine sulfhydrylase A (OASS-A, encoded by *cysK*), and *O*-acetylserine sulfhydrylase B (OASS-B, encoded by *cysM*). The two isoforms are differently expressed under aerobic/anaerobic conditions [22,23] and differ for their substrate selectivity and specificity (OASS-B, but not OASS-A, can use either thiosulfate or sulfide as the sulfur source) and interaction with SAT (not observed with OASS-B) [24,25,26,27]. One of the most interesting studies reported in literature on this subject shows the phenotypic effects of the inhibition of cysteine biosynthesis on antibiotic resistance. Investigations of deletion mutants of this pathway in *Salmonella enterica* serovar Typhimurium led to the results that the inactivation of cysteine biosynthesis, through an unpaired oxidative stress response, causes a reduction in antibiotic resistance in vegetative and swarm cell populations and, as a consequence, conventional antibiotics are effective at lower doses [28,29]. These results highlight the possibility that using cysteine biosynthesis inhibitors could enhance the efficacy of antibiotic treatment, allowing the use of antibiotics at a lower dosage and decreasing the spreading of resistance. Starting from these considerations, it is conceivable that chemical inhibition of OASS isoforms represents a promising approach for the development of antibiotic adjuvants. In recent years, our group and others have developed inhibitors of cysteine biosynthesis in bacteria and mycobacteria for application as enhancers of antibiotic therapy [30,31,32,33,34,35].

**Figure 1:**
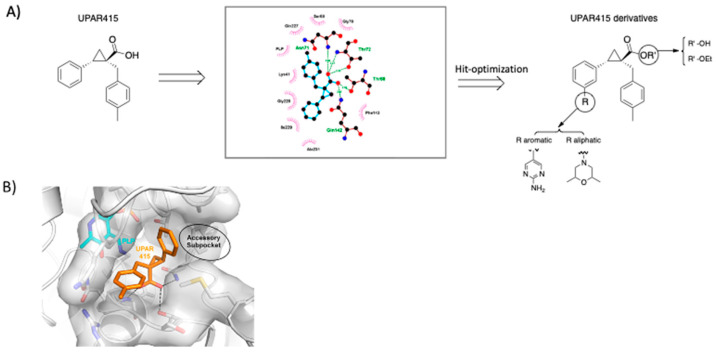
(**A**) Chemical structure of UPAR415, on the left; the main interactions derived from X-ray studies of UPAR415 with the active site are shown in the middle; and the general structure of UPAR415 derivatives are on the right, with the substituents of the most active compounds being shown. (**B**) Cartoon representation of the StOASS-A active site in complex with UPAR415 (PDB code: 6Z4N). Highlighted with a dashed black line is the H-bond interaction of the ligand carboxylic function with the enzyme active site. In close proximity to the pendant phenyl ring, the solvent-exposed accessory sub-pocket targeted by the introduction of the substituent in position 3′ is indicated by a black circle.

In our previous works, we initiated a program directed to the design and synthesis of substituted cyclopropane carboxylic acids, with the aim of exploring their activity toward both OASS-A and OASS-B isoforms. To the best of our knowledge, UPAR415 (Figure 1A) is the most potent inhibitor toward isoforms A and B from *S.* Typhimurium, with a K_D_ of 0.028 μM and 0.490 μM, respectively [36]. In a recent study [37], it was demonstrated that UPAR415 can act as colistin adjuvant with a synergistic or additive effect against all bacterial species considered in that study. Furthermore, it was possible to demonstrate the target engagement in cell by using the *S.* Typhimurium DW378 strain, in which the genes encoding for OASS-A and OASS-B were catalytically inactive [25]. Indeed, the phenotypic effect observed with the use of UPAR415 perfectly overlapped with those observed in the *cysK*- and *cysM*-inactivated *S.* Typhimurium strains, supporting the fact that UPAR415 acts in the cell by the inhibition of OASS-A and -B isoforms. Furthermore, the crystal structure of UPAR415 in complex with OASS-A was resolved by X-ray diffraction studies and confirmed that the UPAR415 binds in the active site and competes with the amino acid substrate. Finally, UPAR415 is able to interfere with SAT binding to OASS-A active site, suggesting that the compound might have effects on the regulation of cysteine biosynthesis not limited to the inhibition of OASS-A activity [38]. Starting from these results, we explored the chance to identify compounds able to selectively target the bacterial RSAP by interfering with OASS isoforms, and to provide a good starting point supporting OASS as potential pharmaceutical target to develop a new class of colistin adjuvants. A medicinal chemistry campaign was initiated with the aim to synthesize UPAR415 derivatives (Figure 1A) in the context of a multiparametric optimization process, since we reported [39] the application of a new integrated approach, which combines enhanced sampling methods with STD experiments for the characterization of ligand-target complexes, as an instrument for drug design purposes. This approach allowed us to consider the ligand–target complex from a dynamic point of view, revealing the presence of an accessory sub-pocket which can be filled and explored by adding different substituents at the 3′ position of the substituted cyclopropane carboxylic acids, in order to obtain novel and more potent StOASS-A inhibitors (Figure 1B). UPAR415 derivatives with a substitution at position 3′ were tested in vitro toward OASS-A and OASS-B both as acids and esters. This choice was dictated by the observation that ester derivatives can behave as pro-drugs, favoring bacterial wall penetration and being hydrolyzed once inside the bacterial cells, thus releasing the active form of the compounds. All acidic derivatives have shown good activities in the nanomolar range against both OASS isoforms in vitro. Minimal Inhibitory Concentrations (MICs) were then evaluated, as well as compounds’ cytotoxicity. The compounds endowed with good activity in vitro and low toxicity have been challenged as a potential colistin adjuvant against different pathogenic bacteria in vitro and the fractional inhibitory concentration (FIC) index has been calculated to define additive or synergistic effects. Finally, the target engagement inside the *S.* Typhimurium cells was confirmed by using a mutant strain in which the OASS enzymes were inactivated.

## 2. Results and Discussion

### 2.1. Chemistry

In order to easily and quickly prepare a small, focused library of derivatives bearing bulkier and aromatic substituents in position 3′, already optimized synthetic strategies have been followed. The easiest strategy to modify UPAR415 in position 3′ is the introduction of another aromatic function by means of a Suzuki coupling reaction. In this way, a small library of derivatives can be easily generated by using different boronic acid fragments. The easiest strategy to introduce aliphatic heterocycles in position 3′ is using another well-described palladium-catalyzed reaction, the Buchwald–Hartwig reaction.

**Scheme 1:**
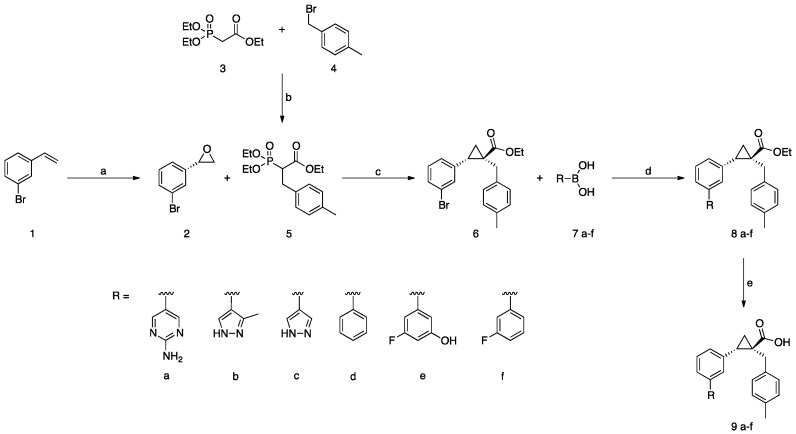
Reagents and conditions for synthesis of compound **8a–f** and **9a–f**: (**a**) m-CPBA, DCM, r.t., 24 h, 86%; (**b**) NaH, DME, r.t. -> 2 h, 60 °C -> 2 h, 56%; (**c**) n-BuLi, DME, r.t., 18 h, 64%; (**d**) Pd(Ph_3_)_4_, K_2_CO_3_, toluene/MeOH/H_2_O (80:18:2), 110 °C, 6 h, 78%; (**e**) LiOH, THF/MeOH/H_2_O (3:1:1), mw, 100 °C, 30′, 56%.

**Scheme 2:**
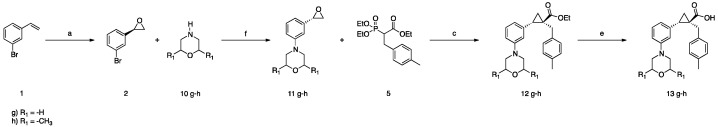
Reagents and conditions for synthesis of compound **12g–h** and **13g–h**: (**a**) m-CPBA, DCM, r.t., 24 h, 86%; (**f**) Pd_2_(dba)_3_, BINAP, NaOtBu, toluene, 100 °C, 18 h, 64%; (**c**) n-BuLi, DME, r.t., 18 h, 64%; (**e**) LiOH, THF/MeOH/H_2_O (3:1:1), mw, 100 °C, 30′, 56%.

**Scheme 3:**
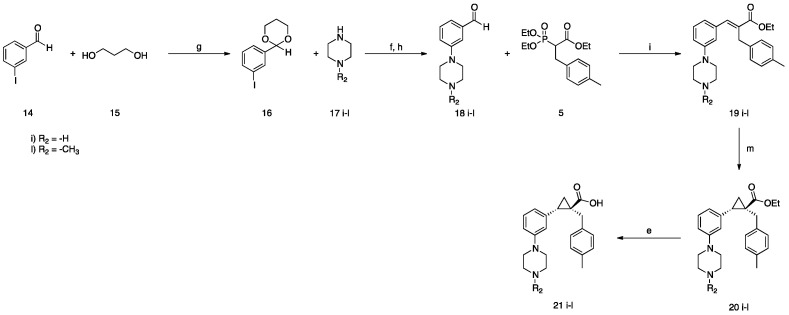
Reagents and conditions for synthesis of compound **20i–j** and **21i–j**: (**g**) p-toluenesulfonic acid, toluene, 110 °C, 18 h, 98%; (**f**) Pd_2_(dba)_3_, BINAP, NaOtBu, toluene, 100 °C, 18 h; (**h**) 1 M HCl, r.t., 3 h, 74%; (**i**) t-BuOK, THF, r.t., 18 h, 67%; (**m**) trimethyl sulfoxonoium iodide, DMSO dry, r.t., 20 h, 72%; (**e**) LiOH, THF/MeOH/H_2_O (3:1:1), mw, 100 °C, 30′, 56%.

For the synthesis of the compounds presented in this work, three different synthetic approaches have been followed. For the synthesis of compounds **8a–f** and **9a–f**, an already published approach optimized in our research laboratory has been followed [25]. Compound 1 was oxidized in presence of m-chloroperbenzoic acid in dichloromethane at room temperature for 18 h to obtain the styrene oxide **2**. The reaction between styrene oxide **2** and the corresponding phosphonate was carried out according to the Wadsworth–Emmons cyclopropanation. In specific, styrene oxide **2**, was treated with the phosphonates **5** in the presence of butyl lithium in dimethoxyethane at 130 °C. The reaction is stereospecific, allowing the desired trans cyclopropane carboxylic ethyl ester **6** to be obtained. Suzuki−Miyaura cross-coupling reaction between the proper boronic acids **7a–f** and compound **6** gave derivatives **8a–f**, which were treated with LiOH at 100 °C under microwave irradiation to afford the desired final compound bearing the carboxylic acid moiety **9a–f** (Scheme 1). For the synthesis of compounds **12g–h** and **13g–h**, we follow a similar synthetic protocol reported in Scheme 1, but in order to add a heteroaliphatic ring at 3′ position of the phenyl ring, a Buckwald–Hartwig reaction [40] was performed in the presence of the appropriate cyclic aliphatic amine **10g–h** and styrene oxide **2** that led to the intermediates **11g–h**. The compounds bearing the ester moiety **12g–h** with the desired trans configuration were obtained through Wadsworth–Emmons cyclopropanation. To obtain the final compounds as carboxylic acids (**13g–h**), basic hydrolysis of the ester moiety was performed in presence of LiOH under microwave irradiation (Scheme 2). For the synthesis of derivatives **20i–j** and **21i–j** (Scheme 3), we have started from compound 3-iodobenzaldehyde (**14**), that has undergone to a protection of the aldehyde group and subsequently to a Buckwald–Hartwig reaction and aldehyde deprotection, leading to intermediates **18i–j**. Horner–Wadsworth–Emmons reaction of these intermediates gave the corresponding acrylates **19i–j**, which have been subjected to cyclopropanation using Corey–Chaykovsky conditions [30], selecting only the compounds in trans configuration **20i–j**. The carboxylic acids **21i–j** have been obtained after hydrolysis of the corresponding ethyl ester (**20i–j**) with LiOH.

### 2.2. Biochemistry

In previous works, our research group reported several small molecules capable of inhibiting StOASS-A and StOASS-B [36,37,38]. Once having rationalized the structure-activity relationship (SAR) profile of reported inhibitors, we looked for robust proof that the evidence derived from our integrated approach could be used to design new StOASS-A inhibitors with a refined pharmacological profile. The presence of an additional accessory pocket previously described and its comparison with X-ray crystal structures so far available for the OASS enzymes revealed that such a pocket is conserved across different orthologues and isoforms and can be occupied by bulkier and hydrophobic substituents.

A series of cyclopropane carboxylic acid derivatives in trans configuration has been synthesized and, to obtain more potent derivatives, the most active compound (UPAR415) has been considered a suitable starting point for further modification. As shown in Table 1, the modifications planned at position 3′ of the phenyl ring allowed one to maintain the trans configuration that is crucial for the activity, but to surmount enantiomeric requirements, as previously described. In this regard, it can be figured out that the introduction of bulkier substituents at the 3′ position can lead to energetically favorable contacts with the protein active site, surmounting the stereochemical requirements. Since the chiral resolution of the racemate or an enantioselective synthesis are no longer required, a significant enhancement in the synthesis of analogs has been reached to expand the series and refine the SAR.

All the compounds have been tested as a racemic mixture and both as carboxylic acids and as the corresponding ethyl ester derivatives (Table 1). As expected, all the derivatives carrying the ester moiety showed no activity in vitro, likely due to the absence of carboxylic moiety responsible for the interaction with the enzyme active site, indicating that the binding mode of these derivatives has not changed. The derivatives bearing the carboxylic moiety showed a good activity toward both StOASS isoforms, from low micromolar to low nanomolar range. Heteroaliphatic and heteroaromatic groups in position 3′ have led to derivatives with improved activity concerning those previously described [39]. All the derivatives can inhibit both OASS isoforms, which is fundamental for cysteine biosynthesis inhibition. Among the derivatives substituted with a heteroaromatic group in 3′ position, compounds **9e** and **9f**, bearing a phenyl substituted with a fluorine and hydroxy group in meta position, or a fluorine, respectively, have shown the most potent inhibitory activities (in the low nanomolar range) toward OASS-A. Compound **8a**, carrying a 2-aminopyrimidine group, has shown a good and comparable in vitro activity toward both isoforms. Compound **13h**, bearing in 3′ position a dimethyl morpholine substituent, has shown the most potent in vitro activity among the derivatives substituted with heteroaliphatic groups in position 3′. Compounds carrying the piperazine ring, **21i** and **21j**, have shown a good activity against OASS-A, but the introduction of these substituents has led to a detrimental effect on the activity against isoform B.

### 2.3. Antimicrobial Activity

After evaluating StOASS enzymes inhibition, the Minimal Inhibitory Concentration (MIC) [41] of each compound alone against the three representative pathogens *Escherichia coli*, *S.* Typhimurium, and *Klebsiella pneumoniae*, belonging to Gram-negative bacteria, has been determined by broth microdilution assay (Table 2). In the Müeller–Hinton Broth (MHB) medium, where cysteine is largely available and its biosynthesis is thus non-essential, OASS is a dispensable enzyme, and its chemical inhibition should not affect bacterial cell growth and survival. For this reason, as expected, the data reported show that all the compounds, used alone, do not possess bactericidal or bacteriostatic effects on the tested strains. Only compound **20i** represents an exception showing a measurable antimicrobial activity, likely due to a strong unspecific cytotoxic activity (vide infra).

### 2.4. Cytotoxicity

**Figure 2:**
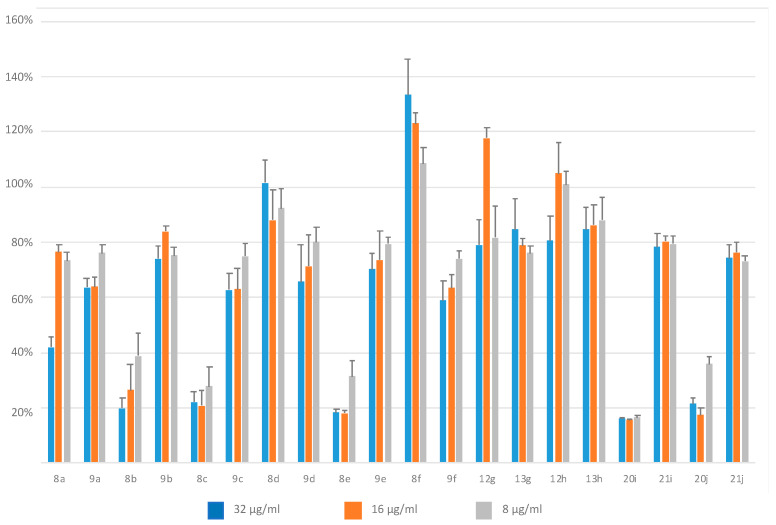
Toxicity evaluation on viability of MDBK cells growing in vitro. On the X-axis, compounds tested; on the Y-axis, percentage of cells viability.

The toxicity of compounds has been evaluated at three different concentrations, 32, 16, 8 µg/mL (Figure 2), through the viability of cells derived from the kidney of adult bovine (MDBK) growing in vitro. The choice of these concentrations was dictated by the fact that since we were looking for compounds with improved adjuvant activities compared to those already reported, we wanted to define the toxic effect of the derivatives at the same concentrations used to test them as colistin adjuvants, lower than the concentrations reported before. Compounds **8b**, **8c**, **8e**, **20i**, and **20j** have shown high toxicity profiles, and for this reason, they were not considered for further investigations. Compounds **8f** and **12g** have shown proliferative effects, highlighting the possibility that they could interact non-specifically with proteins of eukaryotic cells growing in vitro, and they were therefore not considered for further investigations. Compounds **12h** and **13h** have shown the most promising profile, having the lowest toxicity profile, and were advanced to further experiments.

### 2.5. Combination with Colistin

**Figure 3:**
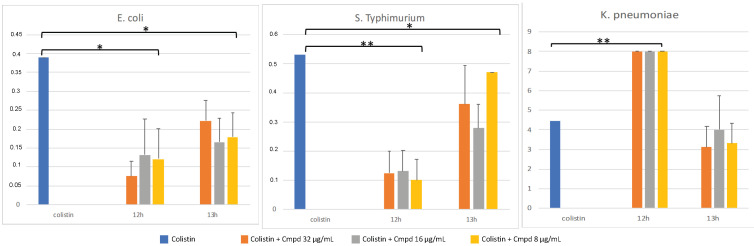
Y-axis: MIC (μg/mL) values of colistin alone or in combination with **12h** and **13h** as adjuvants, in Gram-negative bacteria and in MHB. X-axis: combination of colistin with compounds **12h** and **13h** at different concentrations. Growth was assessed both with unaided eye and using optical density measurements at 620 nm, and percent growth inhibition was calculated in comparison with cells incubated in a medium added with solvent alone (1% DMSO). The results are presented as the average of three independent experiments, each carried out in triplicate, ± standard deviation (* *p* < 0.05, ** *p* < 0.01, between colistin alone and the selected compounds at 8 μg/mL, as determined by ANOVA).

Based on these results, the compounds showing the most promising profile have been advanced to further studies as antibiotic adjuvants. Therefore, compound **13h** and the corresponding ester derivative (**12h**) have been selected for a study in combination with colistin, to evaluate whether the ester moiety could enhance the permeability of this class of compounds. As reported in Figure 3, a remarkable inhibitory effect can be observed on *E. coli* when colistin is used in association with the selected compounds at 8 µg/mL, with a 3- and 2-fold reduction of MIC of colistin for derivatives **13h** and **12h**, respectively. The same trend can be observed in *S.* Typhimurium when the associations of colistin with the selected derivatives at 8 µg/mL are tested. Good results were obtained for derivatives **12h** and **13h** with a 5- and 2-fold reduction with respect to the MIC of colistin alone. These results have shown that the observed effects are higher for derivatives carrying the ester moiety compared to the acid, and are in line with our preliminary hypothesis, suggesting that the ester derivatives better permeate inside the bacterial cell and, once hydrolyzed, release the active form of the compounds. Different results have been obtained for the associations of colistin with derivatives **12h** and **13h** against *K. pneumoniae*. In this case, the association has not led to a reduction of the MIC of colistin and only a slight effect can be observed when colistin is administered with compound **13h** at 32 and 16 µg/mL.

### 2.6. FIC Determination

The FIC index is used to quantitatively define the synergistic interaction between antibiotics and their adjuvants, when two inhibitors are studied in various combinations [42]. A synergistic effect is assumed when the FIC index value is ≤0.5. In Table 3, the FIC index of compounds **12h** and **13h** at 8 μg/mL in association with colistin is reported. When the compounds are tested against *E. coli* and *S.* Typhimurium, the FIC index is consistently lower than 0.5, a value that indicates a synergic interaction. Compound **12h** in association with colistin at 8 μg/mL shows indifference in *K. pneumoniae* while for compound **13h**, the FIC index indicates additive activities in association with colistin against *K. pneumoniae* and *S.* Typhimurium. These observations suggest that using inhibitors of the cysteine biosynthetic pathway as adjuvants in combination with colistin is a promising strategy to fight AMR.

### 2.7. StCysK and CysM Profiling and Comparison with ***12h*** Chemical Inhibition

To prove that the phenotypic effect observed on bacterial cells growing in vitro is linked to the chemical inhibition of OASS-A and/or OASS-B, target engagement experiments have been performed (Table 4). In this regard, compound **12h** has been selected because of its favorable overall properties. It has been tested at a concentration of 32 μg/mL, in order to ensure complete inactivation of both the OASS isoforms. A mutant strain in which *cysK* and *cysM* genes are catalytically inactive [25], the DW378 strain of *S.* Typhimurium, has been used, to prove that the use of **12h** leads to the same phenotypic manifestations observed in the wild type (WT) strain. The MIC assays of **12h** and colistin alone on the DW378 strain have been performed and they have been compared with those obtained on the WT strain. The MIC value of colistin in combination with 32 μg/mL of **12h** in the DW378 strain has been also measured. It was possible to notice that compound **12h** alone does not exert any bactericidal effect on the *Salmonella* DW378 strain, as observed for the WT strain. But, on the other hand, the MIC value of colistin was 5-fold lower on the mutant strain than that on the WT, a result that overlapped those achieved with the association of colistin and **12h** in WT cells. To establish that the synergistic effect observed was due only to the synergystic action of **12h** and colistin in cells, a MIC assay has been performed using **12h** at 32 μg/mL in combination with colistin in the DW378 strain. It is worth noting that there is no change in the colistin MIC in the presence of **12h** in the OASS- inactivated strain. These results perfectly matched with the hypothesis that the effect on bacterial cell viability is mainly due to the synergy of action between colistin and **12h** as OASS inhibitor.

The search of small molecules as colistin adjuvants is a really active field, with intense research activities performed by several groups worldwide supporting the hard interest in this topic, and it is focused on the identification of compounds capable of potentiating its antibiotic and/or restoring its activity against resistant bacteria [43,44]. Albeit, up to now, there have been no drugs approved as colistin adjuvants, and for this reason, the research activity in this field is far from being concluded. In recent years, several colistin adjuvants have been identified, mainly by empirical screening of compound libraries and by rational design of small molecules [45,46,47]. Here, we provide an advancement on a new class of small molecules as colistin adjuvants active in cells, with an unprecedented mechanism of action previously validated by our research groups, acting as inhibitors of cysteine biosynthesis. In this work, we obtained a significant improvement in the active concentration at which our compounds act as colistin adjuvant inside the cells, presenting a follow-up on our research activities on the way to improve pharmacological and biological properties of a new class of small molecules (e.g., multiparametric optimization). Moreover, in the context of a hit-to-lead campaign, the identification of a strategy to disclose prodrugs can pave the way to overcome some limits in colistin adjuvant approach (e.g., toxicity, DMPK issues, combination strategy, and so on), representing a new point of view in the field.

## 3. Materials and Methods

### 3.1. Chemistry

All the reagents were purchased from Sigma-Aldrich (Darmstadt, Germany) and Alfa-Aesar (Haverhill, MA, USA) at reagent purity, and unless otherwise noted, were used without any further purification. Dry solvents used in the reactions were obtained by distillation of technical grade materials over appropriate dehydrating agents. Reactions were monitored by thin layer chromatography on silica gel-coated aluminum foils (silica gel on Al foils, Supelco Analytical, Sigma-Aldrich) at both 254 and 365 nm wavelengths. Where indicated, intermediates and final products were purified through silica gel flash chromatography (silica gel, 0.040−0.063 mm), using appropriate solvent mixtures.

1H NMR and 13C NMR spectra were recorded on a Bruker Advance spectrometer at 400 and 100 MHz, respectively, with TMS as an internal standard. 1H NMR spectra are reported in this order: multiplicity and number of protons. Standard abbreviation indicating the multiplicity was used as follows: s = singlet, d = doublet, dd = doublet of doublets, t = triplet, q = quadruplet, m = multiplet, and br = broad signal. HPLC/MS experiments were performed by a 2695 Alliance separation system (Waters) equipped with a Quattro API tandem quadrupole mass spectrometer (Micromass; Waters, Manchester, UK), using a reversed-phase C18 XSelect^®^ HSS T3 column 2.1 × 50 mm, 2.5 particle size (WATERS, Ireland) see Supplementary Materials. HRMS experiments were performed with an LTQ Orbitrap XL Thermo apparatus (for 1H, 13C spectra and HPLC/MS chromatograms of compounds **8a**, **9a**, **12h**, **13h**, see Supplementary Materials).

All compounds were tested as 95−100% purity samples (by HPLC/MS). 

Synthesis of 2-(3-bromophenyl)oxirane (**2**). Meta-chloroperbenzoic acid (1.25 eq) was added to a solution of 3-bromostyrene (1 eq) in chloroform (3.25 mL/mmol) at 0 °C. The reaction was stirred at room temperature. After 24 h, a TLC showed that the reaction was finished. The reaction mixture was treated with a saturated solution of NaHCO_3_ and extracted with dichloromethane. Then, the organic phases were collected and washed with brine and dried on Na_2_SO_4_. The solvent was removed in vacuum and the crude was purified by flash chromatography using petroleum ether/ethyl acetate starting from 95:5 to 9:1, to obtain a colorless oil as product with 78% yields.

General procedure for the synthesis of oxirane **11 g**–**h**. The appropriate morpholine derivative was added to a stirred suspension of the oxirane 2 in toluene (3 mL/mmol) at room temperature under Argon atmosphere. Then, Pd(dba)_3_, BINAP, and NaOtBu were added. The reaction mixture was heated at 100 °C. After 2 h, a TLC showed that the reaction was finished. The solvent was evaporated and H_2_O was added to the crude and extracted with ethyl acetate. The organic phases were collected and washed with brine and dried over Na_2_SO_4_. The solvent was removed in vacuum and the crude was purified by flash chromatography using dichloromethane/methanol 98:2 as eluent, to obtain the products in yields ranging from 45% to 58%.

General procedure for the synthesis of compound **16**. 1,3-propandiol (0.468 mL, 1.2 eq) and para toluenesulfonic acid (6 mg, 0.006 eq) were added to a solution of 3-iodobenzaldehyde (0.633 mL, 1 eq) in toluene (3 mL/mmol), at room temperature. The reaction mixture was stirred at 110 °C. After 18 h, a TLC showed that the reaction was finished. The solvent was evaporated and H_2_O was added to the crude and extracted with ethyl acetate. The organic phases were collected and washed with brine and dried over Na_2_SO_4_. The solvent was removed in vacuum and the crude was purified by flash chromatography using petroleum ether/ethyl acetate 95:5 as eluent, to obtain the product in 93% yields.

General procedure for the synthesis of compounds **18 i**–**j**. Piperazines **17 i**–**l** (0.178 g, 1.2 eq), Pd(dba)_3_ (0.006 g, 0.005 eq), BINAP (0.014 g, 0.015 eq), 1 M t-BuOK (0.242 g, 1.7 eq) were added to a solution of compound **16** (0.340 g, 1 eq) in toluene (3 mL/mmol), and the resulting mixture was stirred at 100 °C. After 4 h, a TLC showed that the reaction was finished. The reaction mixture was purified through celite and the solvent was removed in vacuum. H_2_O was added to the crude and extracted with ethyl acetate. The organic phases were collected and washed with brine and dried over Na_2_SO_4_. The solvent was removed in vacuum and the crude was dissolved in a solution of 1 M HCl (12 mL) at 0 °C and the resulting mixture was stirred for 2 h at room temperature. A solution of 1 M NaOH was added until pH 11 was reached. The aqueous solution was extracted with ethyl acetate three times. The organic phases were collected and washed with brine and dried over Na_2_SO_4_. The solvent was removed in vacuum and the crude was purified by flash chromatography using dichloromethane/methanol 95:5 as eluent, to obtain the product in 74% yields.

General procedure for the synthesis of compounds **19 i**–**j**. Compound **5** was added at 0 °C, under nitrogen flux, to a solution of 1 M t-BuOK (0.340 g, 1 eq) in dry THF (1 mL/mmol). The mixture was stirred for 30 min and then compound 18 (i-l) was added. The reaction was stirred for 30 min at 0 °C and then at room temperature. After 3 h, a TLC, with dichloromethane/methanol 9:1 as eluent, showed that the starting material was finished. H_2_O was added to the crude and extracted with ethyl acetate. The organic phases were collected and washed with brine and dried over Na_2_SO_4_. The solvent was removed in vacuum and the crude was purified by flash chromatography using petroleum ether/ethyl acetate 95:5 as eluent, to obtain the product in 67% yields.

General procedure for the synthesis of compounds **20i–j**. Trimethyl sulfoxonium iodide (0.047 g, 1.2 eq) was added to a suspension of NaH (0.007 g, 1.2 eq) in DMSO dry at room temperature, under nitrogen flux. The reaction mixture was stirred for 1 h at room temperature and then compound **19(i–l**) was added. The reaction was stirred at room temperature overnight. After 20 h, a TLC, with dichloromethane/methanol 9:1 as eluent, showed that the starting material was finished. H_2_O was added to the crude and extracted with ethyl acetate. The organic phases were collected and washed with brine and dried over Na_2_SO_4_. The solvent was removed in vacuum and the crude was purified by flash chromatography using petroleum ether/ethyl acetate 95:5 as eluent, to obtain the product in 72% yields.

Compounds **5**, **6**, **8a**–**f**, **9a**–**f**, **12g**–**h**, **13g**–**h** and **21i**–**l** have been synthesized as reported by Magalhães et al. [28].

2-(3-bromophenyl)oxirane (**2**) 1H NMR(300 MHz, CDCl3) δ: 7.46–7.44 (m, 2H); 7.28–7.22 (m, 2H); 3.85–3.88 (m, 1H); 3.16 (dd, 1H, J1 = 4, J2 = 5,4); 2.79–2.76 (m, 1H).

HRMS (ESI) calculated for C_8_H_7_BrO ([M-H]-) 196.96801; found 196.96854.

ethyl 2-(3-bromophenyl)-1-(4-methylbenzyl) cyclopropanecarboxylate (**6**) 1H NMR (400 MHz, CDCl_3_) δ: 7.5 (m, 3H); 7.28–7.18 (m, 3H); 7.12–7.02 (m, 5H); 4.16 (t, 2H, J = 7); 3.20–3.16 (m, 1H); 2.84–2.78 (m, 1H); 2.34–2.31 (s, 3H); 2.03–1.97 (m, 1H); 1.93–1.87 (m, 1H); 1.39 (dd, 1H, J1 = 5, J2 = 7).

HRMS (ESI) calculated for C_20_H_21_BrO_2_ ([M-H]-) 371.07254; found 371.07244.

ethyl 2-(3-(2-aminopyrimidin-5-yl)phenyl)-1-(4-methylbenzyl)cyclopropanecarboxylate (**8a**) 1H NMR (400 MHz, DMSO) δ: 8.59 (s, 2H); 7.55–7.49 (m, 2H); 7.39 (t, J = 8, 1H); 7.32 (d, 1H); 7.24–6.99 (m, 4H); 6.78 (s, 2H); 4.07–4.03 (m, 2H); 2.91 (d, 1H); 2.82–2.78 (m, 1H); 2.22 (s, 3H); 2.07 (d, 1H);1.80–1.1.77 (m, 1H); 1.71–1.67 (m, 1H); 1.12(t, J = 4, 3H).

13C NMR (100.6 MHz, DMSO) δ: 173.92; 163.43; 156.53; 137.66; 137.25; 135.73; 135.14; 129.37; 128.96; 128.87; 128.03; 126.51; 124.25; 122.28; 60.84; 33.02; 32.05; 31.44; 21.03; 17.44; 14.44.

HRMS (ESI) calculated for C_24_H_25_N_3_O_2_ ([M-H]-) 386.19873; found 386.19892.

ethyl 2-(3-(3-methyl-1H-pyrazol-4-yl)phenyl)-1-(4-methylbenzyl)cyclopropanecarboxylate (**8b**) 1H NMR (300 MHz, CDCl_3_) δ: 7.52–7.28 (m, 5H); 7.16 (d, 1H, J = 7); 7.05 (s, 4H); 4.22–4.11 (m, 2H); 3.21 (d, 1H, J = 15,5); 2.90 (t, 1H, J = 8); 2.30 (s, 3H); 2.09–2.04 (m,2H); 1.90 (dd, 1H, J1 = 5, J2 = 9); 1.47–1.43 (m, 1H); 1.34–1.26 (m, 4H).

13C NMR (100.6 MHz, CDCl_3_) δ: 169.83; 139.64; 139.31; 136.26; 135.43; 134.91; 132.94; 129.03; 128.81; 127.65; 124.72; 118.65; 61.98; 45.11; 43.01; 42.32; 32.51; 21.32; 14.13; 13.20.

HRMS (ESI) calculated for C_24_H_26_N_2_O_2_ ([M-H]-) 373.19940; found 373.19954.

trans-ethyl 2-(3-(1H-pyrazol-4-yl)phenyl)-1-(4-methylbenzyl)cyclopropanecarboxylate (**8c**) 1H NMR (300 MHz, CDCl_3_) δ: 7.52–7.28 (m, 5H); 7.16 (d, 1H, J = 7); 7.05 (s, 5H); 4.22–4.11 (m, 2H); 3.21 (d, 1H, J = 15.5); 2.90 (t, 1H, J = 8); 2.09–2.04 (m, 2H); 1.90.1.87 (m, 1H); 1.47–1.43 (m, 1H); 1.34–1.26 (m, 4H).

13C NMR (100.6 MHz, CDCl_3_) δ: 168.93; 139.64; 138.71; 135.62; 132.93; 130.82; 129.08; 128.81; 127.63; 124.75; 61.93; 45.17; 43.05; 42.31; 32.54; 21.32; 14.11.

HRMS (ESI) calculated for C_23_H_24_N_2_O_2_ ([M-H]-) 359.18383; found 359.18376.

trans-ethyl 2-([1,1′-biphenyl]-3-yl)-1-(4-methylbenzyl)cyclopropanecarboxylate (**8d**) 1H NMR (300 MHz, CDCl_3_) δ: 7.86 (s, 1H); 7.52–7.41 (m, 6H); 7.18 (d, 2H, J = 7); 6.93 (d, 2H, J = 7); 4.22–4.11 (m, 2H); 3.21 (d, 1H, J = 15.5); 2.98 (d, 1H, J = 15.5); 2.56–2.41 (m, 2H); 2.34 (s, 3H); 2.12 (t, 1H, J = 8); 1.29 (t, 3H, J = 8).

13C NMR (100.6 MHz, CDCl_3_) δ: 169.83; 141.5;4 140.81; 139.62; 135.61; 132.95; 129.28; 129.08; 128.84; 128.03; 127.91; 127.07; 125.16; 61.92; 45.1;3 43.07; 42.32; 32.56; 21.37; 14.11.

HRMS (ESI) calculated for C_26_H_26_O_2_ ([M-H]-) 369.19331; found 369.19367.

trans-ethyl 2-(3′-fluoro-5′-hydroxy-[1,1′-biphenyl]-3-yl)-1-(4-methylbenzyl) cyclopropanecarboxylate (**8e**) 1H NMR (300 MHz, CDCl_3_) δ: 7.86 (s, 1H); 7.52–7.43 (m, 7H); 7.18 (d, 2H, J = 7); 6.93 (d, 2H, J = 7); 5.68 (bs, 1H); 4.21–4.19 (m, 2H); 3.21 (d, 1H, J = 15.5); 2.98 (d, 1H, J = 15.5); 2.56–2.41 (m, 3H); 2.34 (s, 3H); 2.12 (t, 1H, J = 8); 1.29 (t, 3H, J = 8).

13C NMR (100.6 MHz, CDCl3) δ: 175.37; 163.85 (d, ^1^J_CF_ = 245 Hz); 157.48 (d, ^3^J_CF_ = 12 Hz); 143.81 (d, 3JCF = 10 Hz); 140.03; 137.24; 136.85; 135.46; 128.87; 128.83; 128.76; 128.53; 128.21; 125.77; 110.11 (d, ^4^J_CF_ = 3 Hz); 106.39 (d, ^2^J_CF_ = 23 Hz); 102.13 (d, ^2^J_CF_ = 24 Hz); 61.36; 33.30; 32.68; 31.03; 20.97; 18.26; 14.11.

HRMS (ESI) calculated for C_26_H_25_FO_3_ ([M-H]-) 403.17885; found 403.17867.

trans-ethyl 2-(3′-fluoro-[1,1′-biphenyl]-3-yl)-1-(4-methylbenzyl)cyclopropanecarboxylate (**8f**) 1H NMR (300 MHz, CDCl_3_) δ: 7.86 (s, 1H); 7.43–7.26 (m, 7H); 7.18 (d, 2H, J = 7); 6.93 (d, 2H, J = 7); 4.21–4.19 (m, 2H); 3.21 (d, 1H, J = 15.5); 2.98 (d, 1H, J = 15.5); 2.56–2.41 (m, 2H); 2.34 (s, 3H); 2.12 (t, 1H, J = 8); 1.29 (t, 3H, J = 8).

13C NMR (100.6 MHz, DMSO-d6) δ: 173.86; 163.13 (d, ^1^J_CF_ = 243 Hz); 142.89 (d, ^3^J_CF_ = 8 Hz); 139.13; 138.01; 137.50; 134.99; 131.10 (d, ^3^J_CF_ = 8 Hz); 129.33; 129.03; 128.92; 128.76; 128.18; 125.69; 123.31 (d, ^4^J_CF_ = 2 Hz); 114.57 (d, ^2^J_CF_ = 21 Hz); 113.98 (d, ^2^J_CF_ = 23 Hz); 60.86; 33.12; 31.94; 31.14; 21.00; 17.46; 14.44. 

HRMS (ESI) calculated for C_26_H_25_FO_2_ ([M-H]-) 387.18392; found 387.18376.

trans-2-(3-(2-aminopyrimidin-5-yl)phenyl)-1-(4-methylbenzyl)cyclopropanecarboxylic acid (**9a**) 1H NMR (400 MHz, DMSO) δ: 8.59 (s, 2H); 7.52–7.48 (m, 2H); 7.39 (t, J = 8, 1H); 7.23 (d, 1H); 7.05–6.99 (m, 4H); 6.77 (s, 2H); 2.92 (d, 1H); 2.82–2.78 (m, 1H); 2.22 (s, 3H); 2.04 (d, 1H); 1.67–1.63 (m, 2H).

13C NMR (100.6 MHz, DMSO) δ: 175.79; 163.42; 156.51; 138.10; 137.67; 135.68; 134.95; 129.34; 128.93; 128.84; 127.95; 126.52; 124.10; 122.33; 33.05; 31.82; 31.25; 21.03; 17.30.

HRMS (ESI) calculated for C_22_H_21_N_3_O_2_ ([M-H]-) 358.16367; found 358.16343.

trans-2-(3-(3-methyl-1H-pyrazol-4-yl)phenyl)-1-(4-methylbenzyl)cyclopropanecarboxylic acid (**9b**) 1H NMR (300 MHz, CDCl_3_) δ: 11.45 (s, 1H); 7.52–7.28 (m, 5H); 7.16 (d, 1H, J = 7); 7.05 (s, 4H); 3.21 (d, 1H, J = 15,5); 2.90 (t, 1H, J = 8); 2.30 (s, 3H); 2.09–2.04 (m,2H); 1.90 (dd, 1H, J1 = 5, J2 = 9); 1.47–1.43 (m, 1H); 1.34–1.26 (m, 4H).

13C NMR (100.6 MHz, CDCl_3_) δ: 175.93; 139.62; 138.33; 136.28; 135.65; 134.78; 132.94; 129.06; 128.83; 128.16; 128.09; 127.62; 124.74; 118.65; 44.88; 42.31; 42.05; 34.72; 21.33; 21.38.

HRMS (ESI) calculated for C_22_H_22_N_2_O_2_ ([M-H]-) 345.16856; found 345.16812.

trans-2-(3-(1H-pyrazol-4-yl)phenyl)-1-(4-methylbenzyl)cyclopropanecarboxylic acid (**9c**) 1H NMR (300 MHz, CDCl_3_) δ: 11.40 (s, 1H); 7.52–7.28 (m, 5H); 7.16 (d, 1H, J = 7); 7.05 (s, 4H); 3.21 (d, 1H, J = 15.5); 2.90 (t, 1H, J = 8); 2.09–2.04 (m,2H); 1.90 (dd, 1H, J1 = 5, J2 = 9); 1.47–1.43 (m, 1H); 1.34–1.26 (m, 4H).

13C NMR (100.6 MHz, CDCl_3_) δ: 175.92; 139.63; 138.72; 135.66; 132.92; 130.87; 129.03; 128.81; 127.65; 124.74; 44.82; 42.39; 42.01; 34.73; 21.37.

HRMS (ESI) calculated for C_21_H_20_N_2_O_2_ ([M-H]-) 331.67353; found 331.6733.

trans-2-([1,1′-biphenyl]-3-yl)-1-(4-methylbenzyl)cyclopropanecarboxylic acid (**9d**) 1H NMR (300 MHz, CDCl_3_) δ: 11.56 (s, 1H); 7.86 (s, 1H); 7.52–7.41 (m, 7H); 7.18 (d, 2H, J = 7); 6.93 (d, 2H, J = 7); 3.21 (d, 1H, J = 15.5); 2.98 (d, 1H, J = 15.5); 2.56–2.41 (m, 2H); 2.34 (s, 3H); 2.12 (t, 1H, J = 8).

13C NMR (100.6 MHz, CDCl_3_) δ: 175.94; 141.58; 140.85; 139.61; 135.60; 132.93; 129.25; 129.07; 128.82; 128.04; 127.97; 127.61; 127.05; 125.18; 44.83; 42.75; 42.07; 34.71; 21.30.

HRMS (ESI) calculated for C_24_H_22_O_2_ ([M-H]-) 341.16202; found 341.16221.

trans-2-(3′-fluoro-5′-hydroxy-[1,1′-biphenyl]-3-yl)-1-(4-methylbenzyl)cyclopropanecarboxylic acid (**9e**) 1H NMR (300 MHz, CDCl_3_) δ: 11.67 (s, 1H); 7.86 (s, 1H); 7.52–7.43 (m, 7H); 7.18 (d, 2H, J = 7); 6.93 (d, 2H, J = 7); 4.21–4.19 (m, 2H); 3.21 (d, 1H, J = 15.5); 2.98 (d, 1H, J = 15.5); 2.56–2.41 (m, 2H); 2.34 (s, 3H); 2.12 (t, 1H, J = 8).

13C NMR (100.6 MHz, CDCl3) δ: 181.17; 163.86 (d, ^1^J_CF_ = 245 Hz); 157.18 (d, ^3^J_CF_ = 12 Hz); 143.79 (d, ^3^J_CF_ = 10 Hz); 140.01; 136.94; 136.60; 135.60; 128.97; 128.95; 128.74; 128.58; 128.29; 125.93; 110.09 (d, ^4^J_CF_ = 3 Hz); 106.60 (d, ^2^J_CF_ = 22 Hz); 102.20 (d, ^2^J_CF_ = 25 Hz); 33.78; 32.81; 30.76; 21.00; 18.72.

HRMS (ESI) calculated for C_24_H_21_FO_3_ ([M-H]-) 375.14751; found 375.14712.

trans-2-(3′-fluoro-[1,1′-biphenyl]-3-yl)-1-(4-methylbenzyl)cyclopropanecarboxylic acid (**9f**) 1H NMR (300 MHz, CDCl_3_) δ: 11.12 (s, 1H); 7.86 (s, 1H); 7.43–7.26 (m, 8H); 7.18 (d, 2H, J = 7); 6.93 (d, 2H, J = 7); 3.21 (d, 1H, J = 15.5); 2.98 (d, 1H, J = 15.5); 2.56–2.41 (m, 2H); 2.34 (s, 3H); 2.12 (t, 1H, J = 8).

13C NMR (100.6 MHz, DMSO) δ: 175.76; 163.16 (d, ^1^J_CF_ = 243 Hz); 142.92 (d, ^3^J_CF_ = 8 Hz); 139.17; 138.03; 137.54; 135.00; 131.22 (d, ^3^J_CF_ = 8 Hz); 129.34; 129.06; 128.95; 128.80; 128.21; 125.74; 123.34 (d, ^4^J_CF_ = 2 Hz); 114.63 (d, ^2^J_CF_ = 21 Hz); 114.00 (d, ^2^J_CF_ = 23 Hz); 33.13; 31.76; 31.18; 21.00; 17.42. 

HRMS (ESI) calculated for C_24_H_21_FO_2_ ([M-H]-) 359.15264; found 359.15233.

4-(3-(oxiran-2-yl)phenyl)morpholine (**11g**) 1H NMR (300 MHz, CDCl_3_) δ: 7.20–7.18 (m, 2H); 6.81 (s, 1H); 6.70 (d, 1H, J = 8); 3.83 (t, 1H, J = 7); 3.65 (t, 2H, J = 8); 3.18 (t, 2H, J = 8); 2.96–2.71 (m, 3H).

13C NMR (100.6 MHz, CDCl_3_) δ: 150.8; 138.3; 129.5; 125.8; 114.7; 110.2; 66.3; 54.2; 53.3; 50.9.

HRMS (ESI) calculated for C_12_H_15_NO_2_ ([M-H]-) 204.11034; found 204.11011.

2,6-dimethyl-4-(3-(oxiran-2-yl)phenyl)morpholine (**11h**) 1H NMR (300 MHz, CDCl_3_) δ: 7.16–7.09 (m, 3H); 6.81 (s, 1H); 3.83 (t, 1H, J = 8); 3.63–3.57 (m, 2H); 2.96–2.83 (m, 4H); 1.18 (s, 6H).

13C NMR (100.6 MHz, CDCl_3_) δ: 150.8; 138.3; 129.5; 125.8; 114.7; 110.2; 72.8; 68.6; 54.2; 50.9; 19.1.

HRMS (ESI) calculated for C_14_H_19_NO_2_ ([M-H]-) 232.14163; found 232.14177.

trans-ethyl 1-(4-methylbenzyl)-2-(3-morpholinophenyl)cyclopropanecarboxylate (**12g**) 1H NMR (300 MHz, CDCl_3_) δ: 7.19–6.93 (m, 8H); 4.24–4.11 (m, 2H); 3.65 (t, 2H, J = 8); 3.18 (t, 2H, J = 8); 2.96–2.71 (m, 2H); 2.34 (s, 3H); 2.12 (t, 1H, J = 7); 1.29 (t, 3H, J = 8); 0.94–0.69 (m, 2H).

13C NMR (100.6 MHz, CDCl_3_) δ: 169.81; 150.74; 140.02; 135.67; 132.91; 129.44; 128.87; 128.04; 123.55; 117.61; 113.10; 66.37; 61.94; 53.38; 45.12; 42.78; 42.30; 32.57; 21.34; 14.12.

HRMS (ESI) calculated for C_24_H_29_NO_3_ ([M-H]-) 378.21092; found 378.21021.

trans-ethyl 2-(3-(2,6-dimethylmorpholino)phenyl)-1-(4-methylbenzyl)cyclopropanecarboxylate (**12h**) 1H NMR (400 MHz, DMSO) δ: 7.24 (t, J = 8, 1H); 7.05 (s, 4H); 6.84–6.74 (m, 3H); 4.21–4.11 (m, 2H); 3.85–3.80 (m, 2H); 3.48–3.44 (m, 2H); 3.21–3.17 (m, 1H); 2.46–2.40 (m, 1H); 1.98 (d, 1H); 1.87–1.83 (m, 1H); 1.33 (s, 6H); 1.22 (t, J = 8, 3H).

13C NMR (100.6 MHz, DMSO) δ:174.74; 151.04; 137.73; 137.33; 135.23; 129.04; 128.72; 128.58; 120.48; 117.11; 114.43; 71.68; 66.48; 60.83; 54.85; 54.81; 33.05; 33.00; 30.87; 20.99; 19.10; 17.99; 14.15.

HRMS (ESI) calculated for C_26_H_33_NO_3_ ([M-H]-) 406.24604; found 406.24632.

trans-1-(4-methylbenzyl)-2-(3-morpholinophenyl)cyclopropanecarboxylic acid (**13g**) 1H NMR (300 MHz, CDCl_3_) δ: 7.10–6.64 (m, 8H); 3.65 (t, 2H, J = 8); 3.18 (t, 2H, J = 8); 2.96–2.71 (m, 2H); 2.34 (s, 3H); 2.12 (t, 1H, J = 7); 0.94–0.69 (m, 2H).

13C NMR (100.6 MHz, CDCl_3_) δ: 175.91; 150.33; 140.01; 135.65; 132.99; 129.17; 128.88; 128.05; 123.54; 117.61; 113.14; 66.34; 53.37; 44.89; 42.40; 42.09; 34.76; 21.39.

HRMS (ESI) calculated for C_22_H_25_NO_3_ ([M-H]-) 350.18775; found 350.18743.

trans-2-(3-(2,6-dimethylmorpholino)phenyl)-1-(4-methylbenzyl)cyclopropanecarboxylic acid (**13h**) 1H NMR (400 MHz, DMSO) δ: 7.18 (t, J = 8, 1H); 7.04–7.00 (m, 4H); 6.83–6.80 (m, 2H); 6.70–6.68 (d, 1H); 3.70–3.65 (m, 2H); 3.60–3.57 (m, 2H); 2.92 (d, 1H); 2.71 (t, J = 8, 1H); 2.24 (s, 6H); 1.98 (t, J = 8, 1H); 1.61–1.58 (m, 1H); 1.52–1.48 (m, 1H).

13C NMR (100.6 MHz, DMSO-d6) δ: 175.90; 151.12, 137.83, 137.75, 134.96, 129.23, 128.98, 128.81, 119.80, 116.69, 114.08, 71.48, 54.14, 32.90, 32.35, 31.00, 21.05, 19.33, 17.31.

HRMS (ESI) calculated C_24_H_29_NO_3_ ([M-H]-) 288.98042; found 288.98045.

2-(3-iodophenyl)-1,3-dioxane (**16**) 1H NMR (300 MHz, CDCl_3_) δ: 7.84 (s, 1H); 7.23–7.15 (m, 3H); 5.98 (s, 1H); 4.09–3.99 (m, 4H); 1.88–1.85 (m, 2H).

HRMS (ESI) calculated C_10_H_11_IO_2_ ([M-H]-) 288.98042; found 288.98045.

3-(piperazin-1-yl)benzaldehyde (**18i**) 1H NMR (300 MHz, CDCl_3_) δ: 9.88 (s, 1H); 7.46–7.23 (m, 2H); 7.08 (s, 1H); 3.46 (t, 2H, J = 8); 2.86 (t, 2H; J = 8); 

HRMS (ESI) calculated C_12_H_16_N_2_O ([M+H]+) 191.11165; found 191.11132.

3-(4-methylpiperazin-1-yl)benzaldehyde (**18j**) 1H NMR (300 MHz, CDCl_3_) δ: 9.92 (s, 1H); 7.54–7.43 (m, 1H); 7.23 (d, 2H); 7.08 (t, 1H, J = 8); 3.44 (t, 4H, J = 7); 2.36 (t, 4H, J = 7); 2,24 (s, 3H).

HRMS (ESI) calculated C_12_H_16_N_2_O ([M+H]+) 205.14221; found 205.14245.

(E)-ethyl 2-(4-methylbenzyl)-3-(3-(piperazin-1-yl)phenyl)acrylate (**19i**) 1H NMR (300 MHz, CDCl_3_) δ: 7.34 (s, 1H); 7.33 (s, 4H); 7.22–6.89 (m, 3H); 6.57 (s, 1H); 4.71 (bs, 1H); 4.32–4.28 (m,2H); 3.55 (s, 2H); 3.47 (t, 4H, J = 8); 2.83 (t, 4H, J = 8); 2.34 (s, 3H); 1.37 (t, 3H, J = 8).

HRMS (ESI) calculated C_23_H_28_N_2_O_2_ ([M+H]+) 365.23492; found 365.23434.

(E)-ethyl 2-(4-methylbenzyl)-3-(3-(4-methylpiperazin-1-yl)phenyl)acrylate (**19j**) 1H NMR (300 MHz, CDCl_3_) δ:7.31 (s, 1H); 7.24–7.13 (m, 3H); 7.06 (s, 4H); 6.76 (s, 1H); 4.31–4.27 (m, 2H); 3.61 (s, 2H); 3.47 (t, 4H, J = 8); 2.47 (t, 4H, J = 8); 2.31 (s, 3H); 1.34 (t, 3H, J = 7).

HRMS (ESI) calculated C_24_H_30_N_2_O_2_ ([M+H]+) 378.49722; found 379.49715.

trans-ethyl 1-(4-methylbenzyl)-2-(3-(piperazin-1-yl)phenyl)cyclopropanecarboxylate (**20i**) 1H NMR (300 MHz, CDCl_3_) δ: 7.19–6.98 (m, 7H); 6.72 (s, 1H); 4.21 (m, 2H); 3.60 (t, 4H, J = 7); 2.78 (t, 4H, J = 7); 2.63 (s, 2H); 2.37 (s, 3H); 2.12–2.08 (m, 1H); 1.34 (t, 3H, J = 8); 1.13–1.09 (m, 2H).

13C NMR (100.6 MHz, CDCl_3_) δ: 169.76; 150.34; 140.08; 137.89; 132.76; 129.44; 128.78; 127.65; 123.56; 117.87; 114.67; 62.02; 55.03; 54.45; 45.77; 45.17; 42.34; 32.11; 21.76; 17.35.

HRMS (ESI) calculated C_24_H_30_N_2_O_2_ ([M-H]-) 377.23872; found 37.23819.

trans-ethyl 1-(4-methylbenzyl)-2-(3-(4-methylpiperazin-1-yl)phenyl)cyclopropanecarboxylate (**20j**) 1H NMR (300 MHz, CDCl_3_) δ: 7.19–6.98 (m, 7H); 6.72 (s, 1H); 4.21 (m, 2H); 3.60 (t, 4H, J = 7); 2.78 (t, 4H, J = 7); 2.63 (s, 2H); 2.37 (s, 3H); 2.12–2.08 (m, 1H); 1.34 (t, 3H, J = 8); 1.23 (s, 3H); 1.13–1.09 (m, 2H).

13C NMR (100.6 MHz, CDCl_3_) δ: 169.76; 150.34; 140.08; 137.89; 132.76; 129.44; 128.78; 127.65; 123.56; 117.87; 114.67; 62.02; 55.03; 54.45; 45.77; 45.17; 42.34; 32.11; 21.76; 17.35; 15.23.

HRMS (ESI) calculated C_25_H_32_N_2_O_2_ ([M-H]-) 391.25123; found 391.25132.

trans-1-(4-methylbenzyl)-2-(3-(piperazin-1-yl)phenyl)cyclopropanecarboxylic acid (**21i**) 1H NMR (300 MHz, CDCl_3_) δ: 11.09 (bs, 1H); 7.19–6.98 (m, 7H); 6.72 (s, 1H); 4.21 (m, 2H); 3.60 (t, 4H, J = 7); 2.78 (t, 4H, J = 7); 2.37 (s, 3H); 2.12–2.08 (m, 1H); 1.13–1.09 (m, 2H).

13C NMR (100.6 MHz, CDCl_3_) δ: 169.76; 150.34; 140.08; 137.89; 132.76; 129.44; 128.78; 127.65; 123.56; 117.87; 114.67; 62.02; 55.03; 54.45; 45.17; 32.11; 21.76; 17.35.

HRMS (ESI) calculated C_22_H_26_N_2_O_2_ ([M-H]-) 349.77438; found 349.77421.

trans-1-(4-methylbenzyl)-2-(3-(4-methylpiperazin-1-yl)phenyl)cyclopropanecarboxylic acid (**21j**) 1H NMR (300 MHz, CDCl_3_) δ: 11.09 (bs, 1H); 7.19–6.98 (m, 7H); 6.72 (s, 1H); 4.21 (m, 2H); 3.60 (t, 4H, J = 7); 2.63 (s, 2H); 2.12–2.08 (m, 1H); 1.34 (t, 3H, J = 8); 1.23 (s, 3H); 1.13–1.09 (m, 2H).

13C NMR (100.6 MHz, CDCl_3_) δ: 169.76; 150.34; 140.08; 137.89; 132.76; 129.44; 128.78; 127.65; 123.56; 117.87; 114.67; 62.02; 55.03; 54.45; 45.17; 42.34; 32.11; 21.76; 17.35.

HRMS (ESI) calculated C_25_H_32_N_2_O_2_ ([M-H]-) 392.25121; found 391.25132. 

### 3.2. Protein Expression and Purification

All reagents, if not otherwise stated, were purchased from Sigma-Aldrich.

The genes *cysK* and *cysM* coding, respectively, for OASS-A and OASS-B isoforms, were cloned between NdeI and BamHI restriction sites into pET19m vector [35], a modified version of pET19b (Novagen) [48] carrying the sequence coding for the Tobacco Etch Virus (TEV) protease cut site, which enables the remotion of the His-tag from the purified proteins.

Proteins were expressed in *E. coli* Rosetta^TM^ (DE3) cells for 4 h by the addition of 1 mM isopropyl β-D-1-thiogalactopyranoside (Apollo Scientific, Bredbury, UK) during the exponential growth phase. Cells were resuspended in 50 mM NaP, 300 mM NaCl, pH 7.0, in the presence of 1 mg/mL lysozyme, and lysed by sonication. The recombinant proteins were purified by immobilized metal affinity column (IMAC) on cobalt ions (Talon^TM^, Clontech Laboratories, Inc., Mountain View, CA, USA) and were eluted by 250 mM (OASS-A) or 600 mM (OASS-B) imidazole. The His-tag tail was removed by treatment with recombinant TEV protease during O/N dialysis at 4 °C in 10 mM HEPES, pH 8 (OASS-A) or in 20 mM HEPES, 100 mM NaCl, 1 mM tris(2-carboxyethyl)phosphine (TCEP, Apollo Scientific), pH 8.0 (OASS-B). Tag-cleaved proteins were further recovered by separation on IMAC. The proteins showed a purity grade higher than 95% on SDS-PAGE. OASS-A and OASS-B were stored at -80 °C until use in 10 mM HEPES, pH 8.0, and in 20 mM HEPES, 100 mM NaCl, 1 mM TCEP, pH 8.0, respectively.

### 3.3. Determination of Binding Affinity

The affinity of the compounds for StOASS-A and StOASS-B was determined on the purified recombinant proteins by an in vitro fluorescence binding assay [36,49]. Measurements were carried out at 20 °C in a 3 mm pathlength quartz microcuvette in 100 mM HEPES, 5% DMSO (Applichem, Darmstadt, Germany), pH 7.0, using a Fluoromax-4 (Horiba Jobin-Yvon, Kyoto, Japan) spectrofluorometer. Emission spectra of solutions containing 0.2–1.0 μM StOASS, upon excitation of the pyridoxal 5′-phosphate cofactor at 412 nm, in the presence of increasing concentrations of compounds were collected; fluorescence intensities at 500 nm were plotted to compound concentration and data were fitted to a binding isotherm:

$$I=I_{0}+\frac{\Delta I\cdot \left[compound\right]}{K_{D}+\left[compound\right]}$$

where *I* is the fluorescence emission intensity at 500 nm in the presence of a given concentration of compound, *I*_0_ is the initial fluorescence emission in the absence of ligands, Δ*I* is the amplitude of the fluorescence emission change and *K_D_* is the dissociation constant. When the *K_D_* was lower than the protein concentration, data were fitted to the quadratic equation:

$$I=I_{0}+\Delta I\cdot \frac{\left[OASS\right]+\left[compound\right]+K_{D}-\sqrt{{\left(\left[OASS\right]+\left[compound\right]+K_{D}\right)}^{2}-\left(4\cdot \left[OASS\right]\cdot \left[compound\right]\right)}}{2}$$

Data were collected in duplicate.

Esters were tested up to a concentration of 100 μM and no binding was observed.

### 3.4. Evaluation of MIC by Broth Microdilution Assays

MIC values were evaluated following CLSI guidelines with some modifications, as referred to in previous work [41]. Twofold dilutions 25.6–0.05 mg/mL of the tested compounds were performed in DMSO in separate 96-well microtiter U-plates (Greiner, Milan, Italy). In a different 96-well microtiter U-plate, for each well of the replicates, 49 µL of broth medium were added. Then, in each well of the plate, one microliter of each compound at variable dilution in DMSO was added. For each test, three independent experiments with three replicates each were performed.

Tested reference bacterial strains (*E. coli* ATCC25922; *S.* Typhimurium ATCC14028; *K. pneumoniae* ATCC13883) were brought to the logarithmic phase of growth in MHB medium by incubation at 37 °C for 24 h. After incubation, bacterial suspensions were centrifuged (2000 rpm, 4 °C for 20 min), and then the pellets were resuspended in 100 mM phosphate buffer, pH 7.0, to reach a final bacterial concentration of 10^8^ CFU/mL, adjusted by spectrophotometry (OD_620nm_ ranged between 0.08–0.13). The obtained suspensions were further diluted 1:100 in broth medium to reach a bacterial concentration of 10^6^ CFU/mL. Fifty µL of the bacterial suspension containing 106 CFU/mL were inoculated into each well within 30 min, to obtain a final concentration of 5 × 10^5^ CFU/mL in a total volume of 100 µL. The final dilution range tested was 256–0.5 µg/mL. Growth and sterility controls were performed for each strain and for each tested compound. Plates were then incubated for 24 h at 37 °C in a static aerobic atmosphere. After incubation, plates were read by unaided eye with a microtiter reading mirror and then the OD of each well of the plates was measured spectrophotometrically at 620 nm. MIC values were calculated as the arithmetic mean ± standard deviation (SD) of the unaided eye reading and the inhibition of growth for each tested dilution was calculated from the OD values (mean ± SD). A quality control microorganism (*E. coli* ATCC 25922) was tested periodically to validate the accuracy of the procedure.

### 3.5. Checkerboard Assays

Antimicrobial activity of associations between the different compounds and colistin (Sigma-Aldrich, St. Louis, MO, USA, lot n. 049M-4836 V) was tested with checkerboard assay with minor modifications, as mentioned in the previous work [37]. For each assay, three experiments with three replicates each were assessed. For each compound tested concentration, 96-wells microtiter U-plates were prepared with twofold serial dilutions of colistin in MHB starting from the MIC value (µg/mL) for ten consecutive dilutions in 50 µL of broth. In each well of the same replicate, 1 µL of each compound in DMSO was added at a fixed concentration, 100 times higher than the final desired concentration (3.2; 1.6; 0.8 mg/mL). Subsequently, 49 µL of the bacterial suspension at a concentration of 10^6^ CFU/mL, adjusted spectrophotometrically as reported above, was added to each well, reaching the final bacterial concentration of 5 × 10^5^ CFU/mL. Growth and sterility controls were performed for each experiment and for each replicate. Finally, the plates were incubated at 37 °C in aerobic atmosphere for 24 h. After incubation, plates were read by unaided eye with a microtiter reading mirror and then the OD of each well of the plates was measured spectrophotometrically at 620 nm. MIC values were calculated as the arithmetic mean ± SD of the unaided eye reading and the inhibition of growth for each tested dilution was calculated from the OD values (mean ± SD).

To evaluate the antimicrobial effect of the two molecules in association, the FIC index was calculated as follows. The MICs of each of the two molecules tested individually and in combination with each other were evaluated and the results have been included in the following formula as reported by Meletiadis et al. [50]:

$$\mathrm{FIC}=\frac{{\mathrm{MIC}}_{A\;\mathrm{in}\;\mathrm{combination}}}{{\mathrm{MIC}}_{A}}+\frac{{\mathrm{MIC}}_{B\;\mathrm{in}\;\mathrm{combination}}}{{\mathrm{MIC}}_{B}}$$

where MIC_A_ was the MIC of the compound UPAR415 and MIC_A_ in combination was the MIC of UPAR415 in combination with colistin. MIC_B_ was the MIC of colistin and MIC_B_ in combination was the MIC of colistin in combination with UPAR415. From the results of the FIC index formula, the antimicrobial activity in combination of the two molecules can be considered: synergistic, additive, indifferent of antagonistic. If the FIC index is ≤ 0.5, the association is synergic; additive if FIC is between 0.5 and 1; indifferent if FIC is between 1 and 4; and antagonistic if FIC is ≥4 [50].

Statistical analysis of checkerboard results in comparison with MIC values of colistin alone was performed using the Analysis of Variance (ANOVA) test.

### 3.6. Cytotoxicity Assay on MDBK Cells

The cytotoxicity assay was performed using the 3-(4,5-dimethylthiazol-2-yl)-2,5-diphenyltetrazolium bromide (MTT) test, following the procedure used by Donofrio et al. [51]. MDBK cells were cultured on microtiter tissue culture plates in DMEM medium for 24 h at 37 °C in the presence of 5% CO_2_. After incubation, when the cell monolayer was at confluence, an aliquot of 1 µL of UPAR415 in DMSO at different concentrations was added in each well, containing a volume of 100 µL of DMEM medium and MDBK cells, and then the plates were reincubated at the same conditions. After incubation, 10 µL of MTT at 200 mg/mL (3-(4,5-dimethylthiazol-2-yl)-2,5-diphenyltetrazolium bromide) was added to each well and incubated at 37 °C for 6 h. At the end of the incubation, 100 µL of the solubilization solution (10% SDS in 0.01 M HCl) was added to each well and then incubated overnight. The MTT compound, a yellow tetrazolium salt, is reduced by mitochondrial enzymes (succinate dehydrogenase) of metabolically active eukaryotic cells to insoluble formazan crystals. In the presence of metabolically active cells, after the addition of a detergent solution (10% SDS in sterile PBS) that allows the formazan to be released from the cells, a violet color is seen in the medium. Instead, in the presence of non-viable cells, MTT is not reduced to formazan and therefore the solution will remain yellow.

After incubation, plates were read with a spectrophotometer at 620 nm. Positive controls (PC)—without any compounds—and PCs with 1% DMSO were performed for each plate and three replicates for two independent experiments were performed for each assay.

#### Tested Bacterial Strains

Bacterial strains tested in this work were:

- *Escherichia coli* ATCC 25922
- *Salmonella enterica* subsp. Enterica serovar Typhimurium ATCC 14028
- *Klebsiella pneumoniae* ATCC 13883
- *Salmonella enterica* subsp. Enterica serovar Typhimurium DW378 *

* This strain is defective for the expression of *cysK* and *cysM* genes, encoding for isozymes OASS-A and B. The genotypes of this strain are *trpC109*, *cysK1772*, and *cysM1770*. Due to its mutation, DW378 is auxotroph for cysteine and L-tryptophan and resistant to azaserine (TK181). The strain was identified by Hulanicka et al. [25] by isolation of strains lacking OASS-B in the genetic background of TK181 strain lacking OASS-A. In this paper, it was reported that DW378 completely lacks *O*-acetylserine sulfhydrylase activity, but no molecular evidence has been provided for the origin of this phenotype [16]. In our previous work [37], we demonstrated that OASS-A is expressed in comparable amounts both in *S.* Typhimurium ATCC 14028 and in DW378, while OASS-B expression is undetectable in both the strains. Therefore, the cysteine auxotrophy in DW378 is probably due to the secondary inactivation of the enzyme but not to the complete deletion of the encoding genes.

## 4. Conclusions

The use of antibiotic adjuvants could represent a winning strategy to fight AMR. In this context bacterial metabolic pathways, such as cysteine biosynthesis, may represent a target of great interest. *O*-acetylserine sulfhydrylase catalyzes the last step of the cysteine biosynthetic pathway, and since mammals are not equipped with this enzyme, its specific inhibitors would be highly selective and safe. On the basis of the results from our previous efforts, we have rationally designed and synthesized a series of UPAR415 derivatives to be tested against StOASS-A and StOASS-B. Most of the compounds synthesized were found to maintain good binding affinity in the biochemical assays, corroborating the rational design of the analogues and allowing the additional body of SAR to be reported. The toxicity profiles have been evaluated showing that almost all compounds are endowed with good tolerability by MDBK cells. The derivatives showing the best activity in vitro, combined to a good toxicity profile, have been tested as colistin adjuvants revealing strong synergistic or additive effects against *E. coli* and *S.* Typhymurium, even at the lower concentration used (8 μg/mL). Moreover, the most promising derivative (12h) of that series has been used to confirm the target engagement inside bacterial cells. Using *S.* Typhimurium DW378 strain, in which the *cysK* and *cysM* genes were inactivated, has revealed that the phenotypic effects due to the chemical inhibition of the enzymes are overlapping those observed in the mutant strain, confirming what was observed with the hit compound, UPAR415. In this work, we report: (i) the synthesis of 20 new compounds; (ii) their in-vitro and in-cell characterization as colistin adjuvants; (iii) their toxicity profiles; (iv) the validation of MoA of our series of compounds, as stated in our hypothesis; and (v) a strategy to disclose prodrugs that can be instrumental in the context of the multiparametric optimization process, moving forward our class of colistin adjuvants. Finally, the aim of the paper is the identification of the colistin adjuvant, a useful strategy aimed at improving the therapeutic window of colistin on those strains already susceptible. What we wanted to highlight with this work is the possibility to improve the therapeutic window of colistin, and likely widen its spectrum of bacterial strains on which it is not active per se, such as gram-positive bacteria that are insensitive to colistin. Aspects like this were already highlighted in our previous work [37] and are now the object of further investigations also for this series of compounds.

Altogether, these findings provide a solid base to further investigate the use of cysteine biosynthesis inhibitors as antimicrobial adjuvants, since they represent valuable tools able to tune the events associated with bacterial virulence and drug resistance.

## 5. Patent

This work is a part of a research project that led to the approval of a first patent (n. 102019000011412).

## Figures and Tables

**Table 1:** Chemical structure of derivatives **8a**, **9a**, **8b**, **9b**, **8c**, **9c**, **8d**, **9d**, **8e**, **9e**, **8f**, **9f**, **12g**, **13g**, **12h**, **13h**, **20i**, **21i**, **20j**, **21j** and their dissociation constants for StOASS isoforms.

Compound	Structure	K_D_ (μM)	
		OASS-A	OASS-B
**8a**	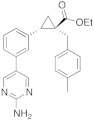	No binding	No binding
**9a**	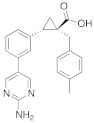	0.57 ± 0.16	1.29 ± 0.09
**8b**	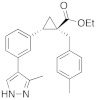	No binding	No binding
**9b**	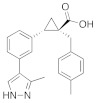	1.86 ± 0.76	2.63 ± 0.04
**8c**	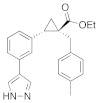	No binding	No binding
**9c**	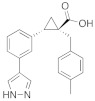	1.01 ± 0.38	3.05 ± 0.18
**8d**	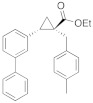	No binding	No binding
**9d**	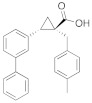	2.51 ± 0.61	6.8 ± 1.1
**8e**	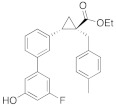	No binding	No binding
**9e**	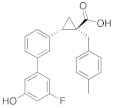	0.035 ± 0.003	0.61 ± 0.08
**8f**	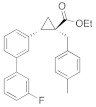	No binding	No binding
**9f**	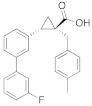	0.051 ± 0.004	1.45 ± 0.31
**12g**	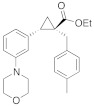	No binding	No binding
**13g**	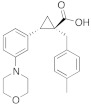	0.10 ± 0.01	3.86 ± 0.29
**12h**	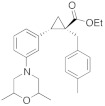	No binding	No binding
**13h**	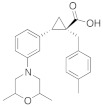	0.066 ± 0.005	3.37 ± 0.72
**20i**	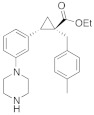	No binding	No binding
**21i**	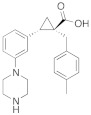	0.45 ± 0.09	83.8 ± 16.1
**20j**	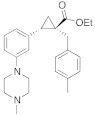	No binding	No binding
**21j**	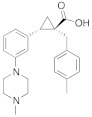	0.25 ± 0.06	23.6 ± 4.5

**Table 2:** Minimal Inhibitory Concentration (MIC) of derivatives tested alone on the Gram-negative bacteria *E. coli*, *S.* Typhimurium, and *K. pneumoniae*.

Compound	MIC (μg/mL)		
	*E.coli* ATCC 25922	*S.* Typhimurium ATCC14028	*K. pneumoniae* ATCC13883
**8a**	>512	>512	>512
**9a**	>512	>512	>512
**8b**	>512	>512	>512
**9b**	>512	>512	>512
**8c**	>512	>512	>512
**9c**	>512	>512	>512
**8d**	>512	>512	>512
**9d**	>512	>512	>512
**8e**	>512	>512	>512
**9e**	>512	>512	>512
**8f**	>512	>512	>512
**9f**	>512	>512	>512
**12g**	>512	>512	>512
**13g**	>512	>512	>512
**12h**	>512	>512	>512
**13h**	>512	>512	>512
**20i**	40	36	32
**21i**	>512	>512	>512
**20j**	>512	>512	>512
**21j**	>512	>512	>512

**Table 3:** FIC index of most potent combinations of compounds **12h** and **13h** with colistin.

Compound (8 μg/mL)	MIC Colistin			FIC Index		
	*E. coli*	*S.* Typhimurium	*K. pneumoniae*	*E. coli*	*S.* Typhimurium	*K. pneumoniae*
-	0.39 ± 0.25	0.53 ± 0.19	4.00 ± 1.73	-	-	-
**12h**	0.122 ± 0.08	0.101 ± 0.07	8.00 ± 0.0	0.33 (synergy)	0.21 (synergy)	2.01 (indifference)
**13h**	0.18 ± 0.06	0.468 ± 0.01	3.33 ± 1.0	0.48 (synergy)	0.90 (additivity)	0.85 (additivity)

**Table 4:** Compound **12h** tested in combination with colistin in *S.* Typhimurium wild type and mutant strains.

Bacterial Strain	MIC 12h (μg/mL)	MIC Colistin (μg/mL)	12h (μg/mL)	MIC Colistin (μg/mL)
*S.* Typhimurium ATCC14028	>512	0.53 ± 0.19	32	0.125 ± 0.08
*S.* Typhimurium DW378	>512	0.141 ± 0.09	-	0.141 ± 0.09
			32	0.141 ± 0.09

## Data Availability

The data presented in this study are available in this article.

## Supplementary Materials

The following are available online at https://www.mdpi.com/article/10.3390/ph15060766/s1, Figure S1: Ethyl 2-(3-(2-aminopyrimidin-5-yl)phenyl)-1-(4-methylbenzyl)cyclopropane-1-carboxylate (**8a**), 1H NMR (400 MHz, DMSO); Figure S2: Ethyl 2-(3-(2-aminopyrimidin-5-yl)phenyl)-1-(4-methylbenzyl)cyclopropane-1-carboxylate (**8a**), 13C NMR (100.6 MHz, DMSO; Figure S3: HPLC/MS analysis (**8a**) Method A; Figure S4: 2-(3-(2-aminopyrimidin-5-yl)phenyl)-1-(4-methylbenzyl)cyclopropane-1-carboxylic acid (**9a**) 1H NMR (400 MHz, DMSO); Figure S5: 2-(3-(2-aminopyrimidin-5-yl)phenyl)-1-(4-methylbenzyl)cyclopropane-1-carboxylic acid (**9a**), 13C NMR (100.6 MHz, DMSO); Figure S6: HPLC/MS analysis (**9a**) Method A; Figure S7: Ethyl 2-(3-(2,6-dimethylmorpholino)phenyl)-1-(4-methylbenzyl)cyclopropane-1-carboxylate (**12h**), 1H NMR (400 MHz, CDCl_3_); Figure S8: Ethyl 2-(3-(2,6-dimethylmorpholino)phenyl)-1-(4-methylbenzyl)cyclopropane-1-carboxylate (**12h**), 13C NMR (400 MHz, CDCl_3_); Figure S9: HPLC/MS analysis (**12h**) Method B; Figure S10: 2-(3-(2,6-dimethylmorpholino)phenyl)-1-(4-methylbenzyl)cyclopropane-1-carboxylic acid (**13h**), 1H NMR (400 MHz, DMS); Figure S11: 2-(3-(2,6-dimethylmorpholino)phenyl)-1-(4-methylbenzyl)cyclopropane-1-carboxylic acid (**13h**), 13C NMR (100.6 MHz, DMSO); Figure S12: HPLC/MS analysis (**13h**) Method B.
