# Supplementary material for: Inhibitors of O-Acetylserine Sulfhydrylase with a Cyclopropane-Carboxylic Acid Scaffold Are Effective Colistin Adjuvants in Gram Negative Bacteria
Source: Pharmaceuticals (Basel). 2022 Jun 20;15(6):766. doi: 10.3390/ph15060766 (PMC9227781; doi:10.3390/ph15060766)

Inhibitors of *O*-acetylserine sulfhydrylase with a cyclopropane-carboxylic acid scaffold are effective colistin adjuvants in Gram negative bacteria.

Giannamaria Annunziato[a]*, Costanza Spadini[b], Marialaura Marchetti[c], Nina Franko[d], Marialaura Pavone[a], Mattia Iannarelli[b], Agostino Bruno[a], Marco Pieroni[a], Stefano Bettati[c], Clotilde Silvia Cabassi[b], Barbara Campanini[d], Gabriele Costantino[a].

SUPPORTING INFORMATION

Analytical methods (HPLC-ESI-MS)

Samples were injected (10 µL) and chromatographically separated using a reversed-phase C18 XSelect® HSS T3 column 2.1 x 50 mm, 2.5 particle size (WATERS, Ireland). A gradient profile was applied using water (eluent A) and acetonitrile (eluent B), acidified with 0.2% and 0.1 % formic acid respectively, as a mobile phase. Mass spectrometry data were collected in positive electrospray mode over the range of m/z 100-600. Source settings were maintained using a capillary voltage of 2.5 kV (compounds **8a** and **9a**), 3.5 kV (compound **12h**), or 4.0 kV (compound **13h**); a cone of 25 V (compounds **8a** and **9a**) or 30 (compounds **12h** and **13h**), source temperature, 120°C; desolvation temperature, 350 °C, and desolvation gas flow, 700 L/h.

Method A: Initial conditions were set up at 5% of B, after 2.0 min of isocratic step, a linear change to 100% of B in 8.0 min. 100% B was achieved in 10 min and held for 5 min to allow for the column washing before returning to the initial conditions. Column recondition was achieved over 5 min, proving a total run time of 20 min. The column was maintained at 30°C and a flow rate of 0.200 mL/min was used.

Method B: The gradient started with 100% of A, after 2.0 min of isocratic step, a linear change to 90% of B in 8.0 min. 100% B was achieved in 10 min and held for 5 min to allow for the column washing before returning to the initial conditions. Column recondition was achieved over 1 min and held for 7 min, proving a total run time of 23 min. The column was maintained at 30°C and a flow rate of 0.200 mL/min was used.

Figure S1.

Ethyl 2-(3-(2-aminopyrimidin-5-yl)phenyl)-1-(4-methylbenzyl)cyclopropane-1-carboxylate (8a)

^{1}H NMR (400 MHz, DMSO-d_6)

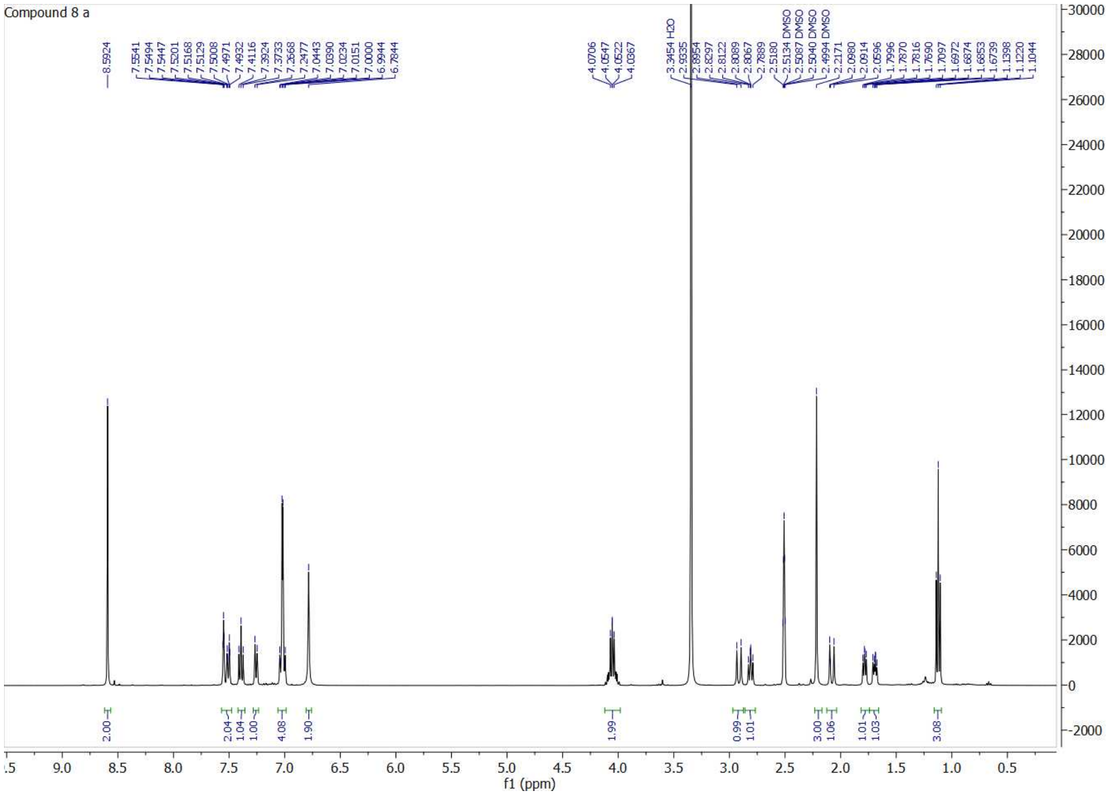

Figure S2.

Ethyl 2-(3-(2-aminopyrimidin-5-yl)phenyl)-1-(4-methylbenzyl)cyclopropane-1-carboxylate (8a)

^{13}C NMR (100.6 MHz, DMSO-d_6)

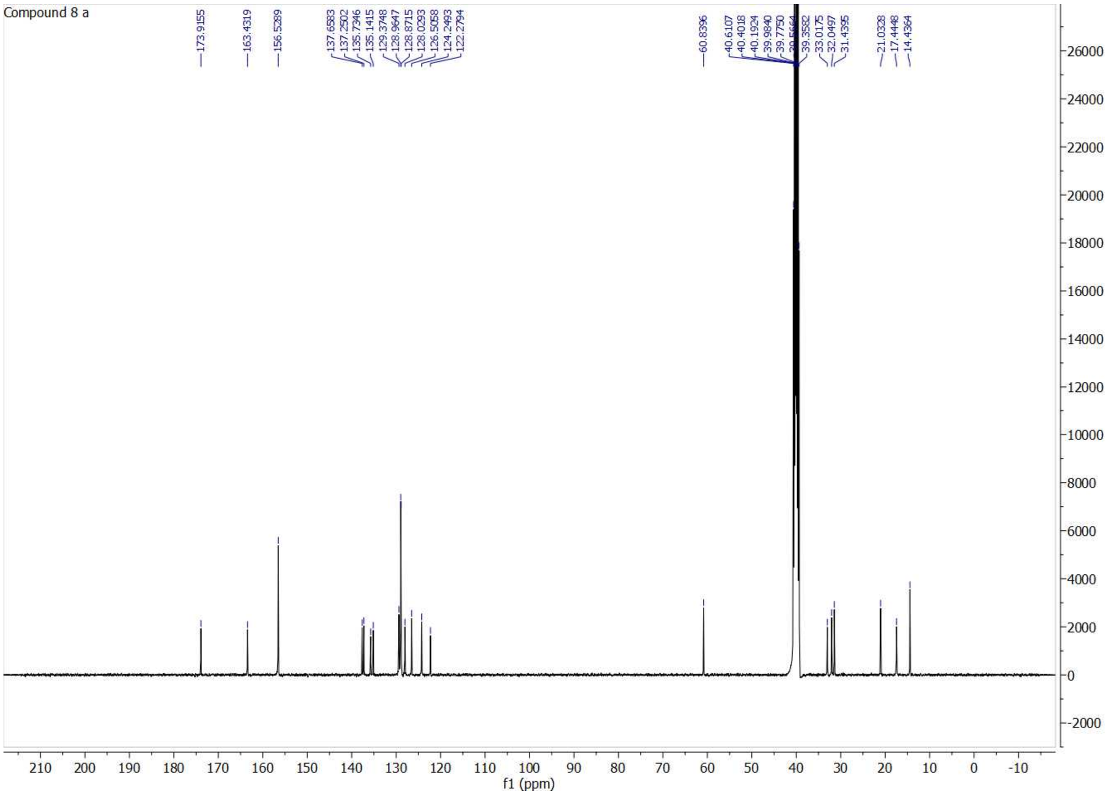

Figure S3.

HPLC/MS analysis (8a)

Method A

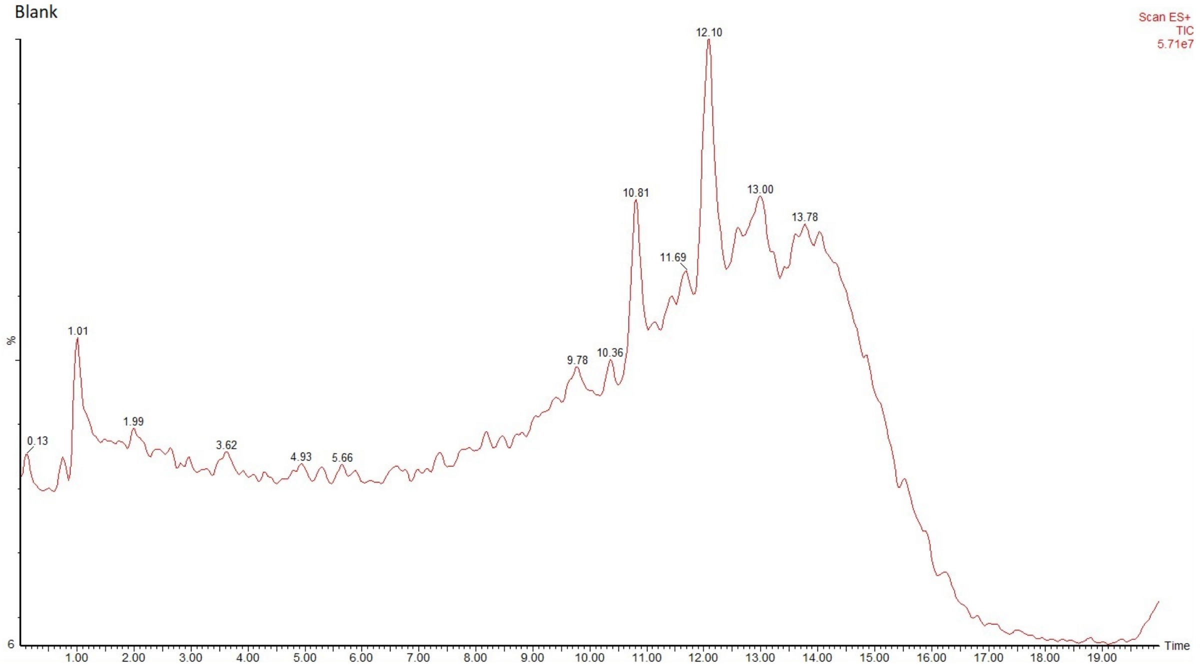

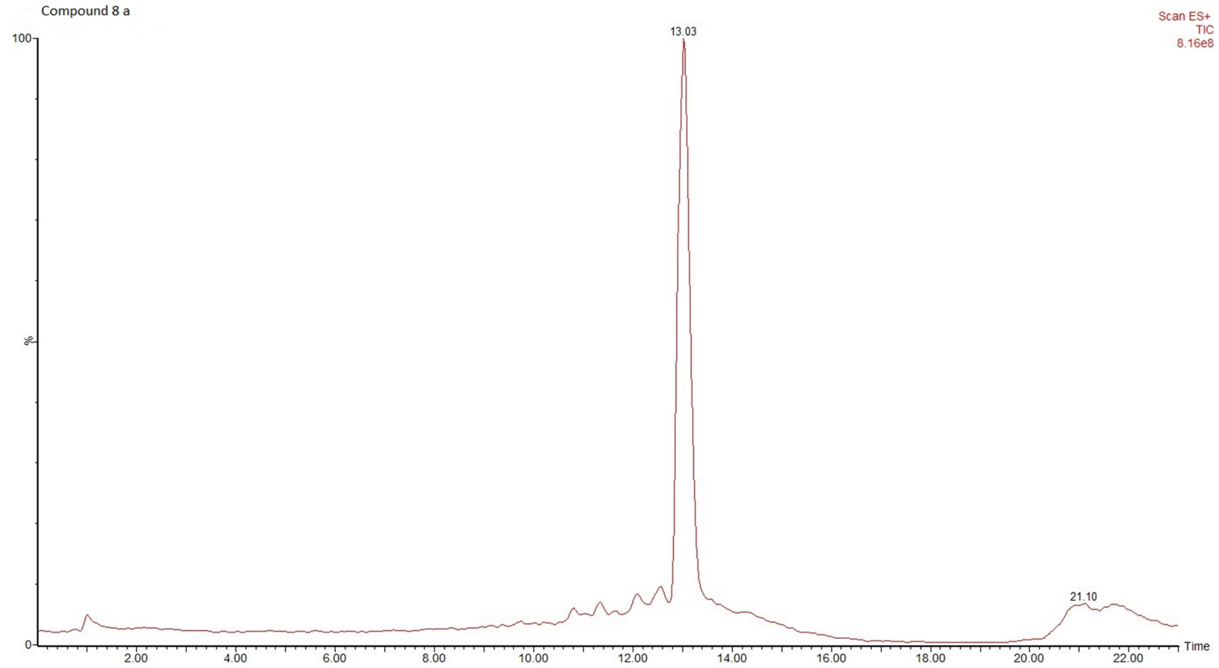
Compound 8 a
Scan ES+
TIC
8.16e8
13.03
21.10
100
%
0
2.00
4.00
6.00
8.00
10.00
12.00
14.00
16.00
18.00
20.00
22.00
Time

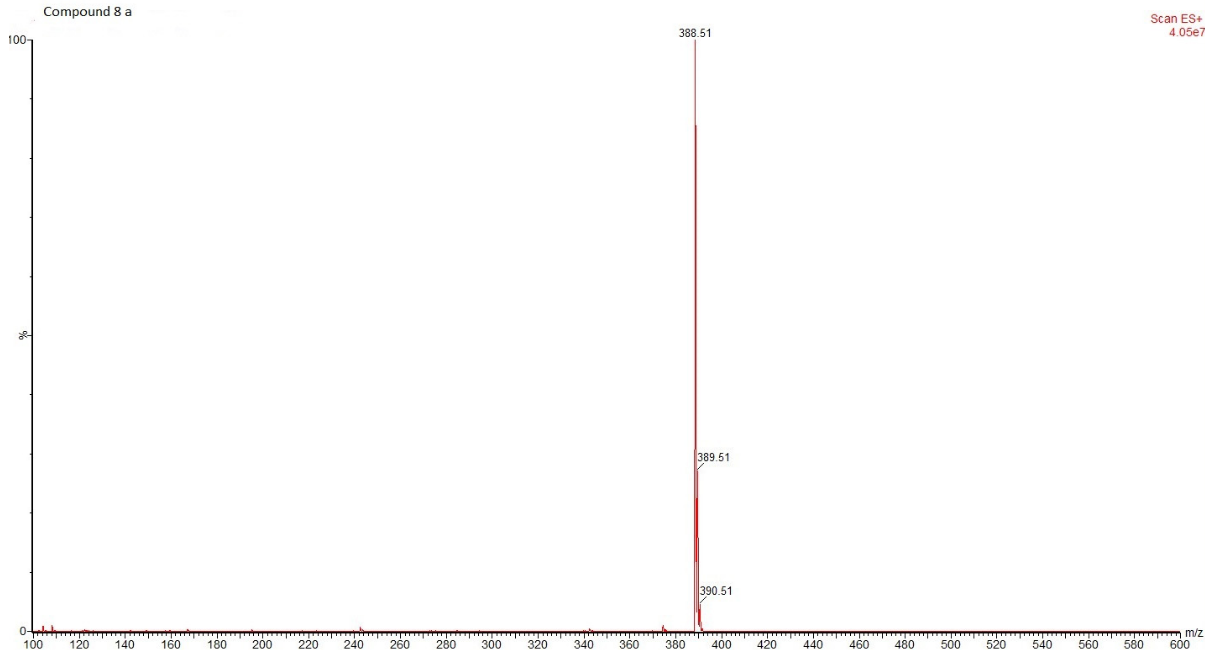
Compound 8 a
Scan ES+
4.05e7
388.51
389.51
390.51
100
%
0
100
120
140
160
180
200
220
240
260
280
300
320
340
360
380
400
420
440
460
480
500
520
540
560
580
600
m/z

Figure S4.

2-(3-(2-aminopyrimidin-5-yl)phenyl)-1-(4-methylbenzyl)cyclopropane-1-carboxylic acid (9a)

^{1}H NMR (400 MHz, DMSO-d_6)

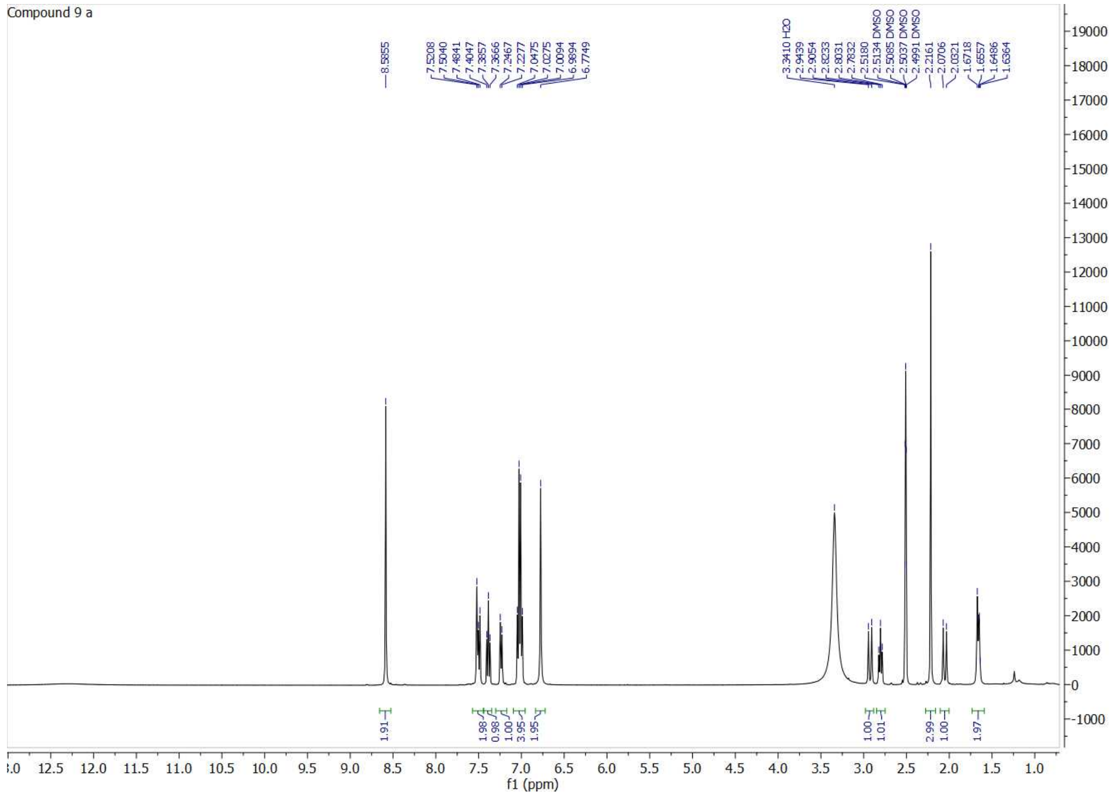

Figure S5.

2-(3-(2-aminopyrimidin-5-yl)phenyl)-1-(4-methylbenzyl)cyclopropane-1-carboxylic acid (9a)

^{13}C NMR (100.6 MHz, DMSO-d_6)

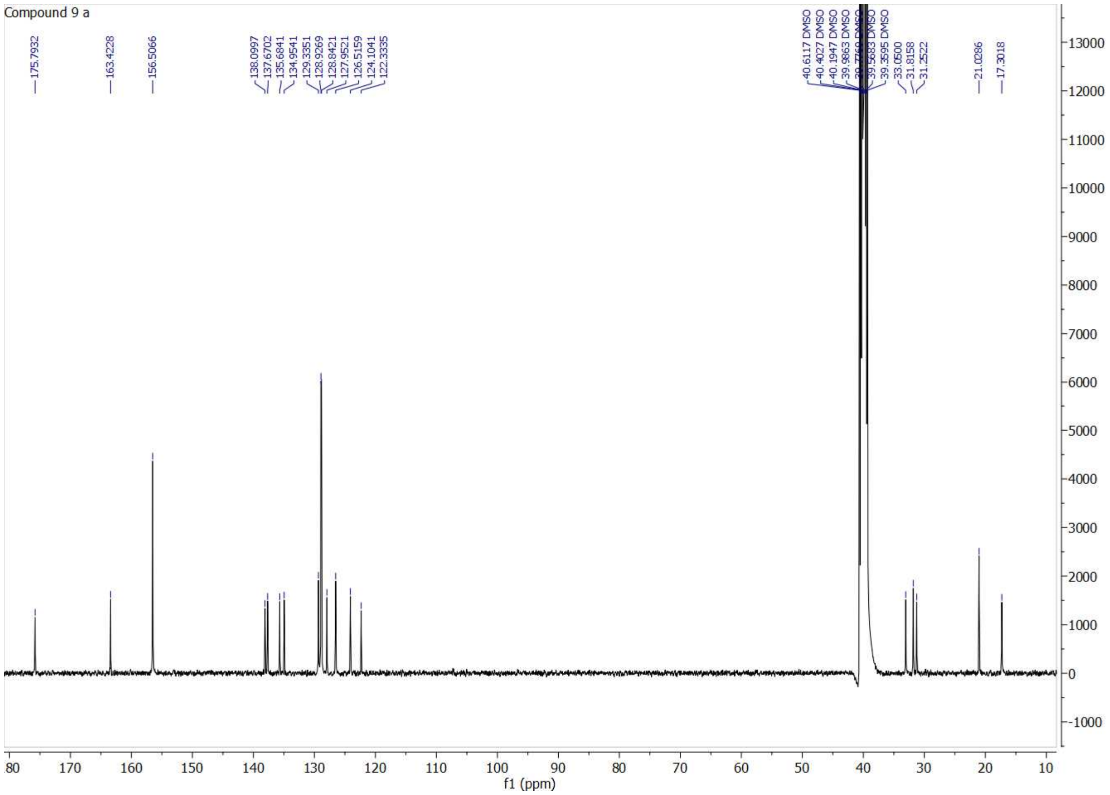

Figure S6.

HPLC/MS analysis (9a)

Method A

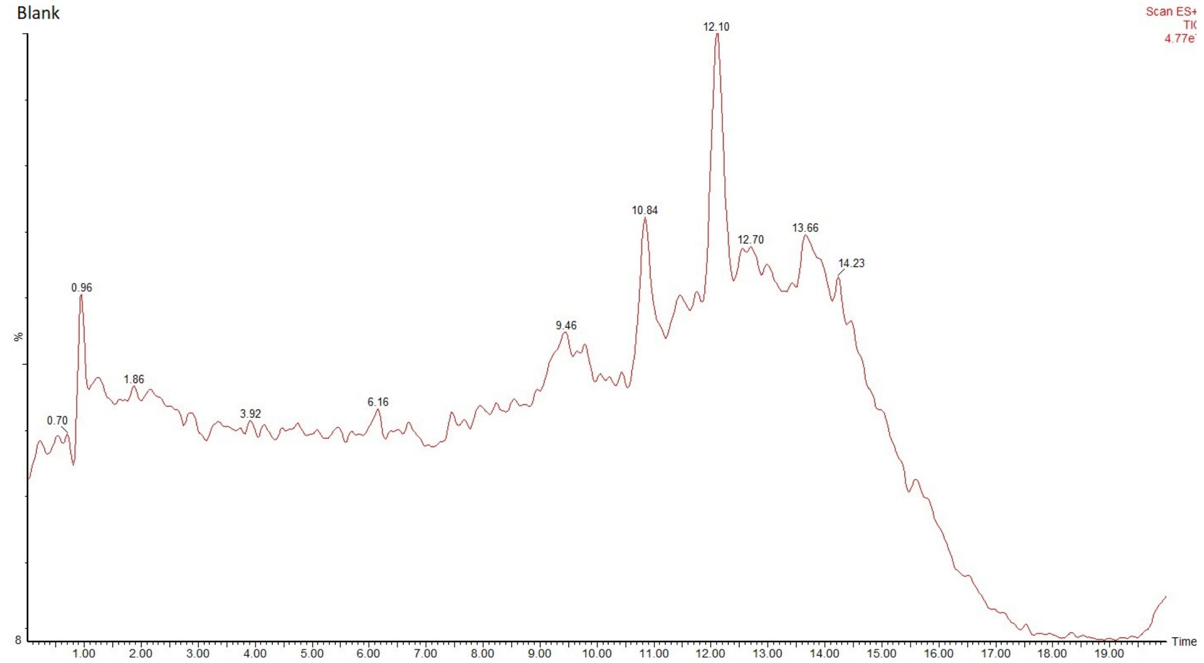

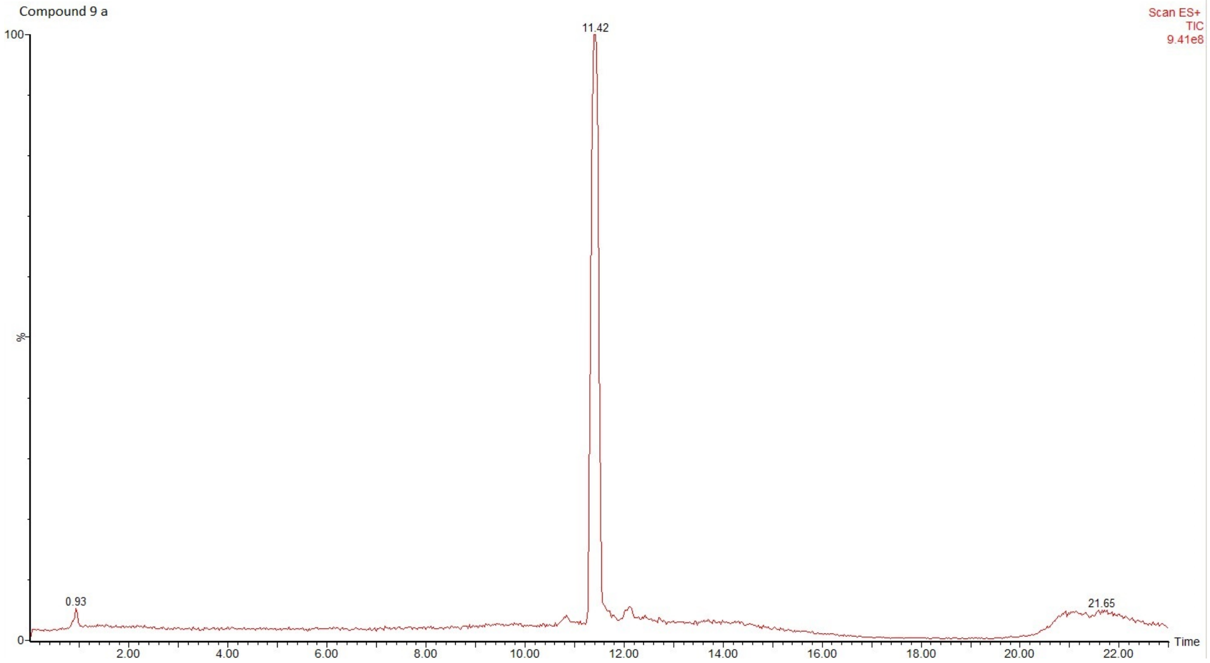

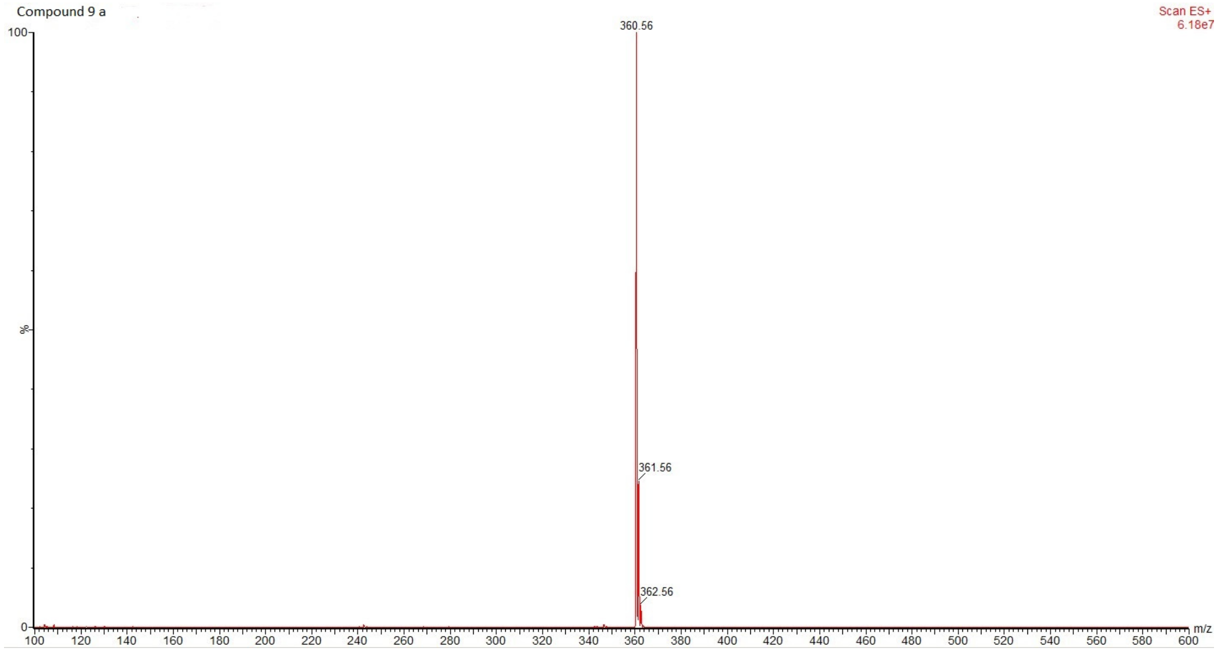

Figure S7.

Ethyl 2-(3-(2,6-dimethylmorpholino)phenyl)-1-(4-methylbenzyl)cyclopropane-1-carboxylate (12h)

^1H NMR (400 MHz, $CDCl_3$)

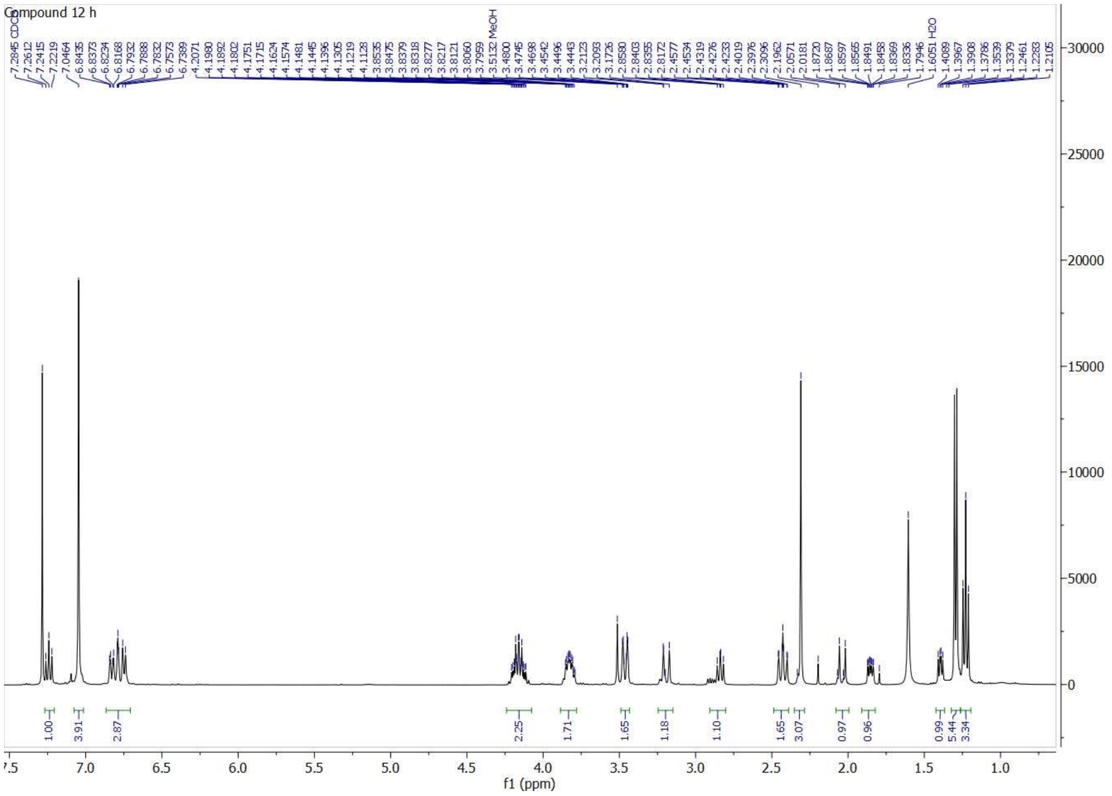

Figure S8.

Ethyl 2-(3-(2,6-dimethylmorpholino)phenyl)-1-(4-methylbenzyl)cyclopropane-1-carboxylate (12h)

^{13}C NMR (400 MHz, $CDCl_3$)

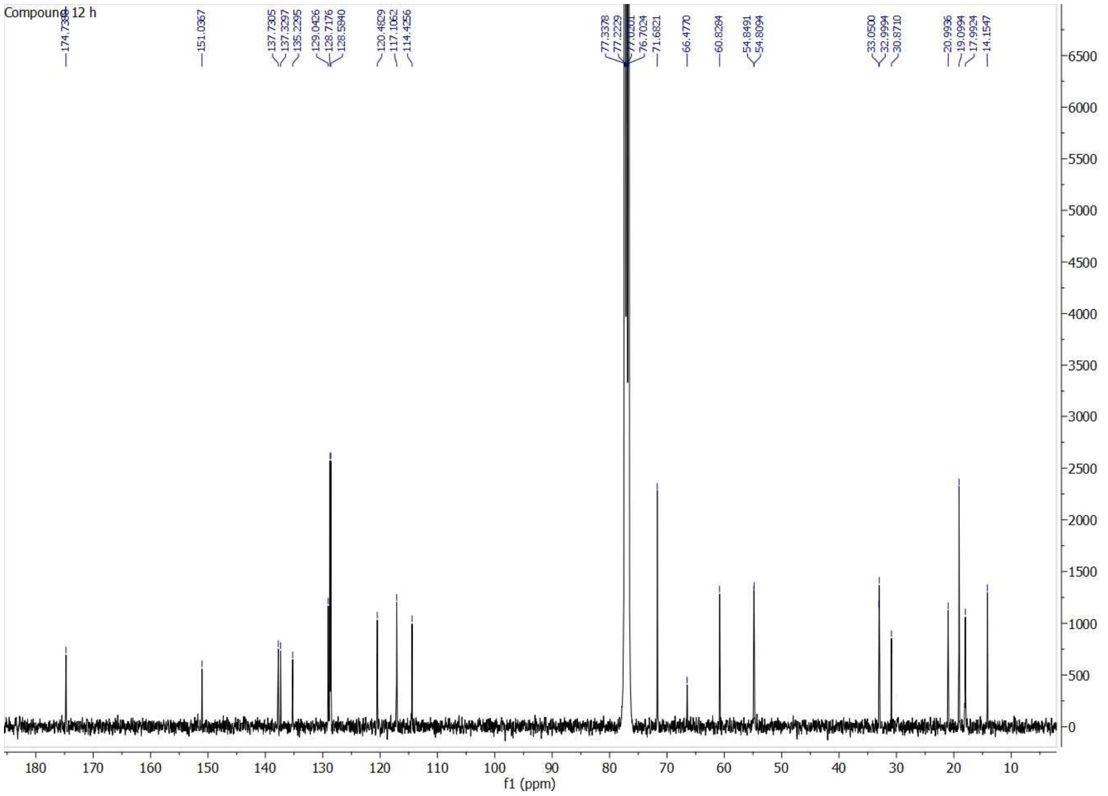

Figure S9.

HPLC/MS analysis (12h)

Method B

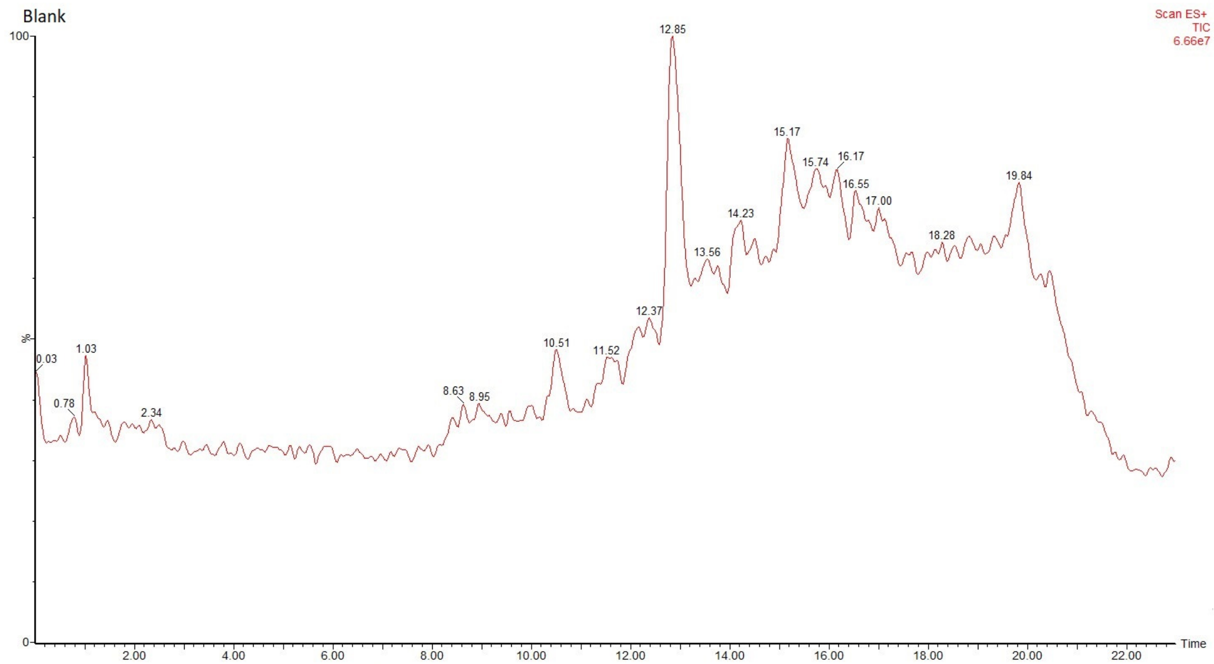

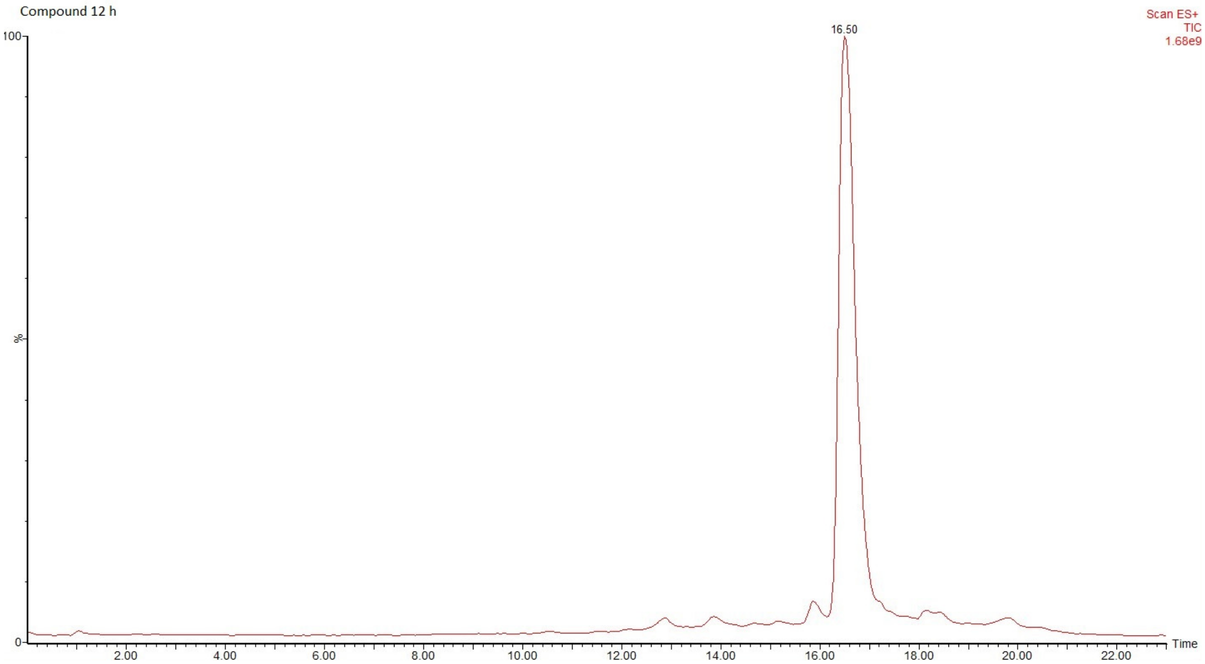

Compound 12 h

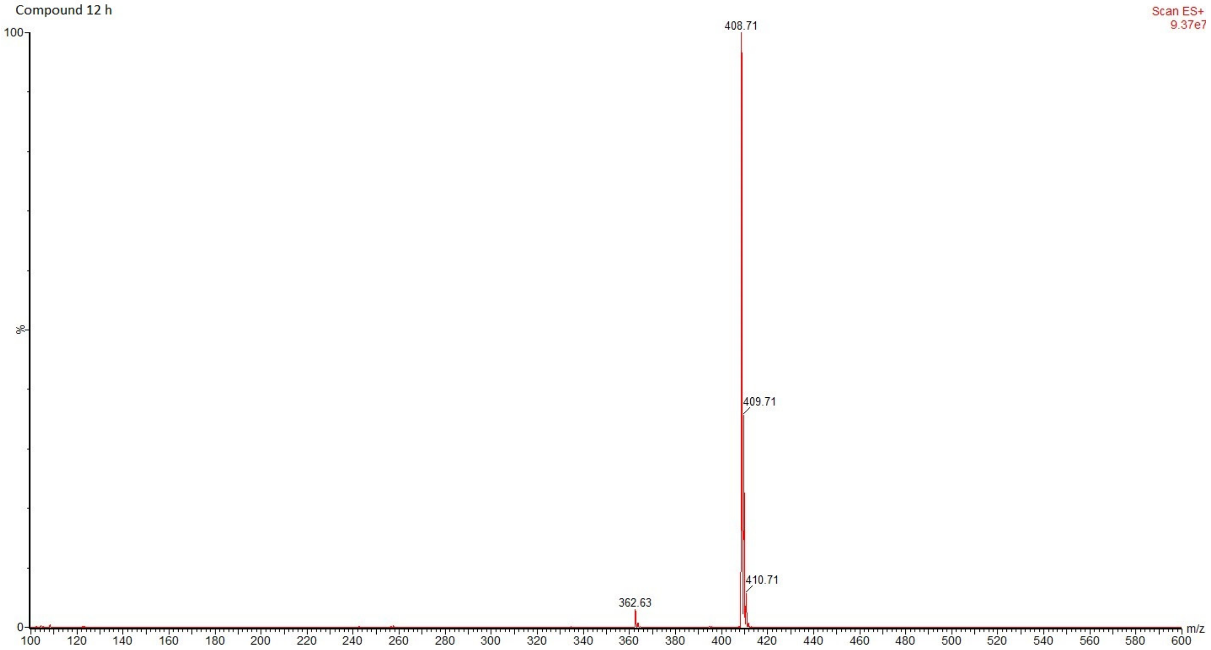

Figure S10.

2-(3-(2,6-dimethylmorpholino)phenyl)-1-(4-methylbenzyl)cyclopropane-1-carboxylic acid (13 h)

^{1}H NMR (400 MHz, DMSO-d_6)

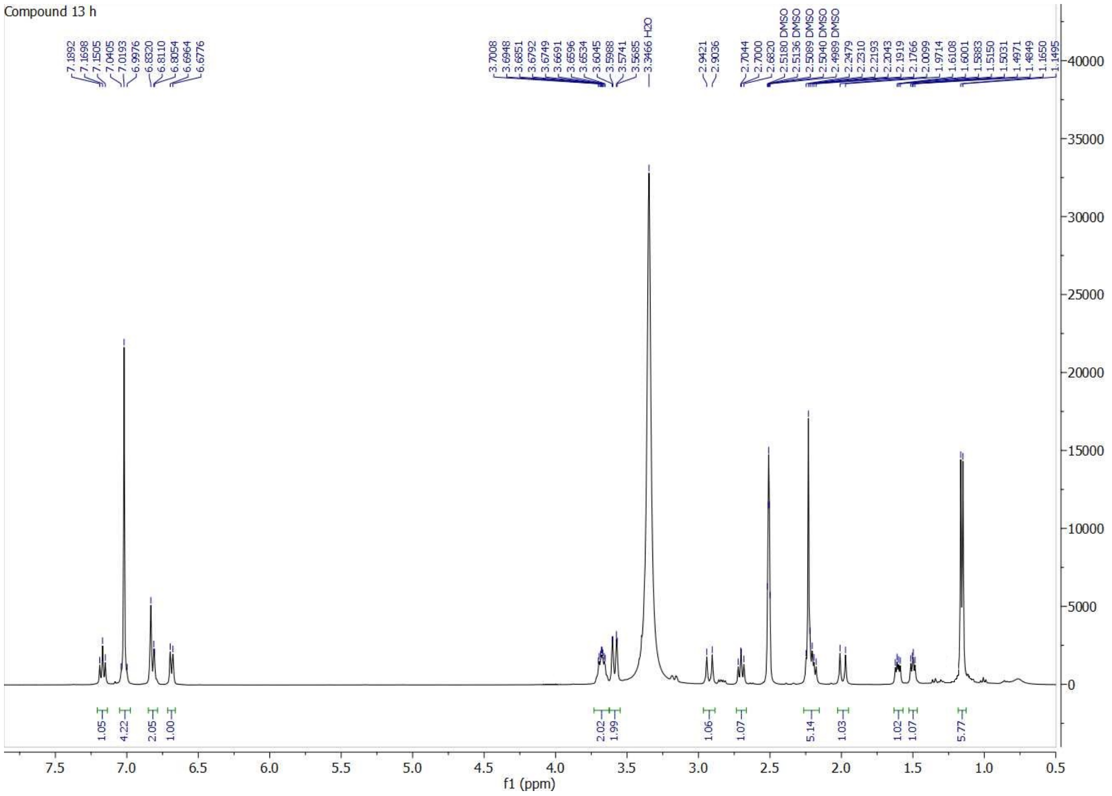

Figure S11.

2-(3-(2,6-dimethylmorpholino)phenyl)-1-(4-methylbenzyl)cyclopropane-1-carboxylic acid (13 h)

^{13}C NMR (100.6 MHz, DMSO-d_6)

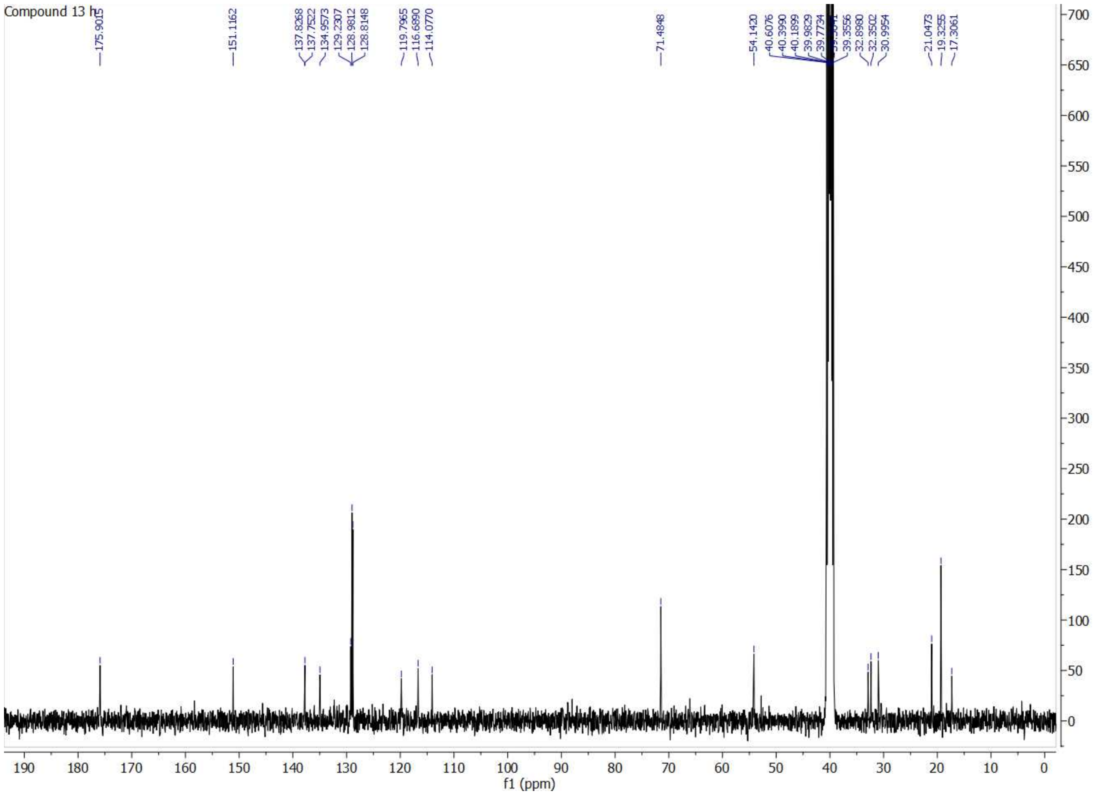

Figure S12.

HPLC/MS analysis (13h)

Method B

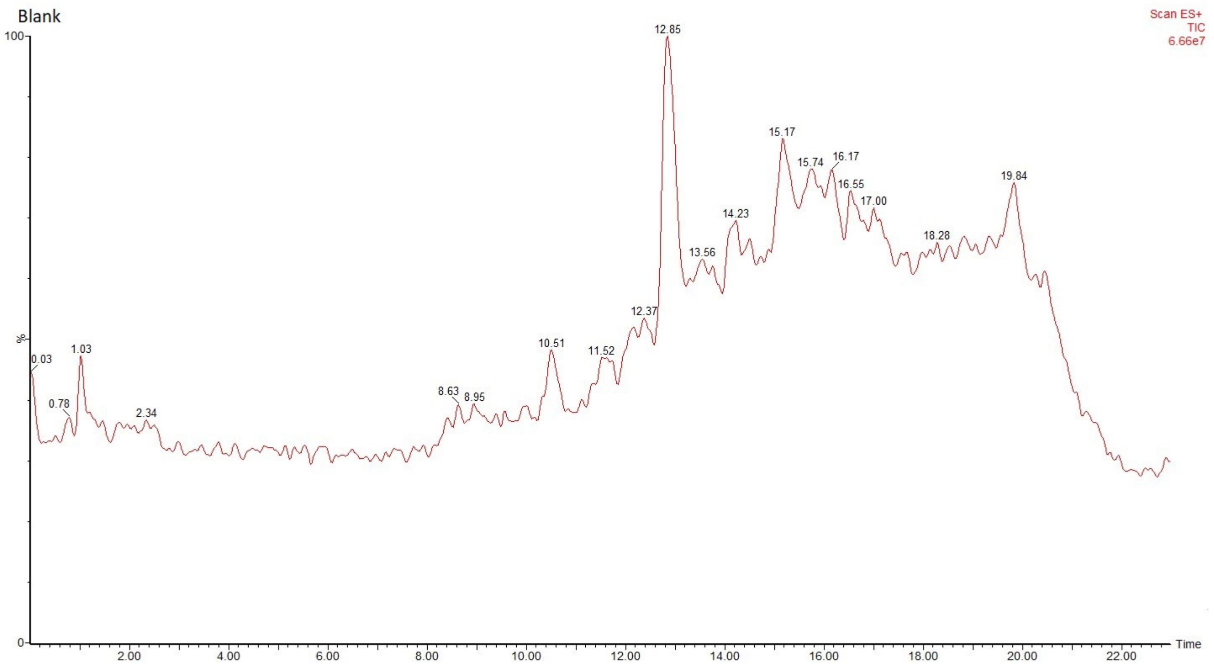

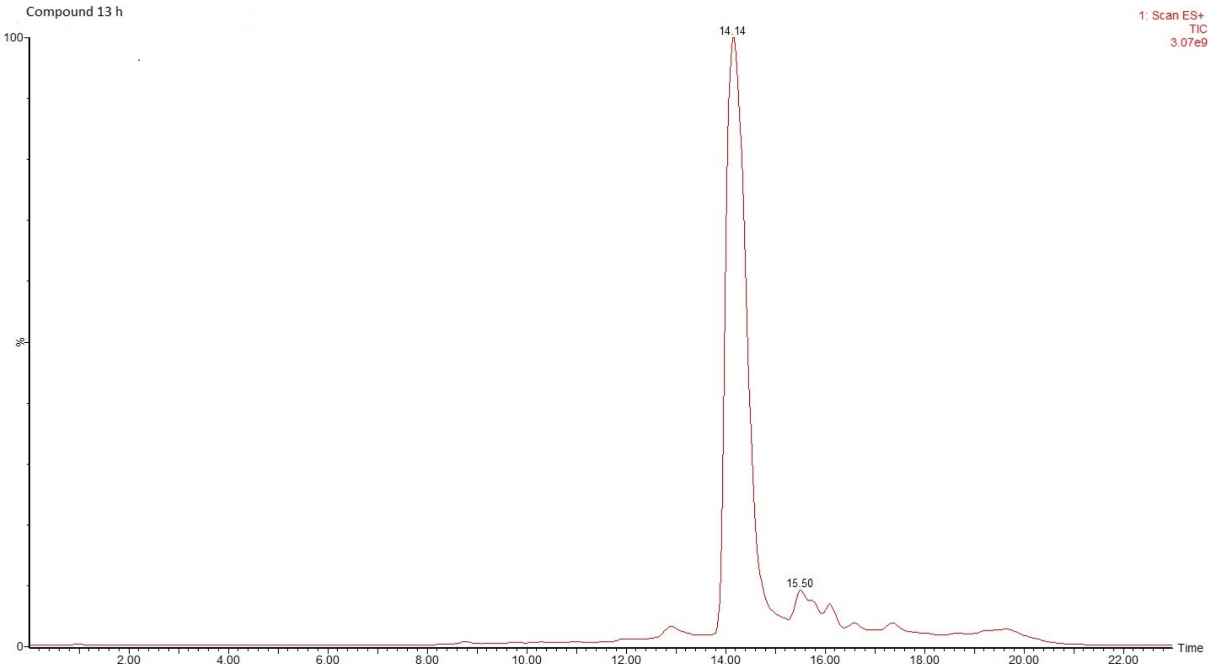

Compound 13 h

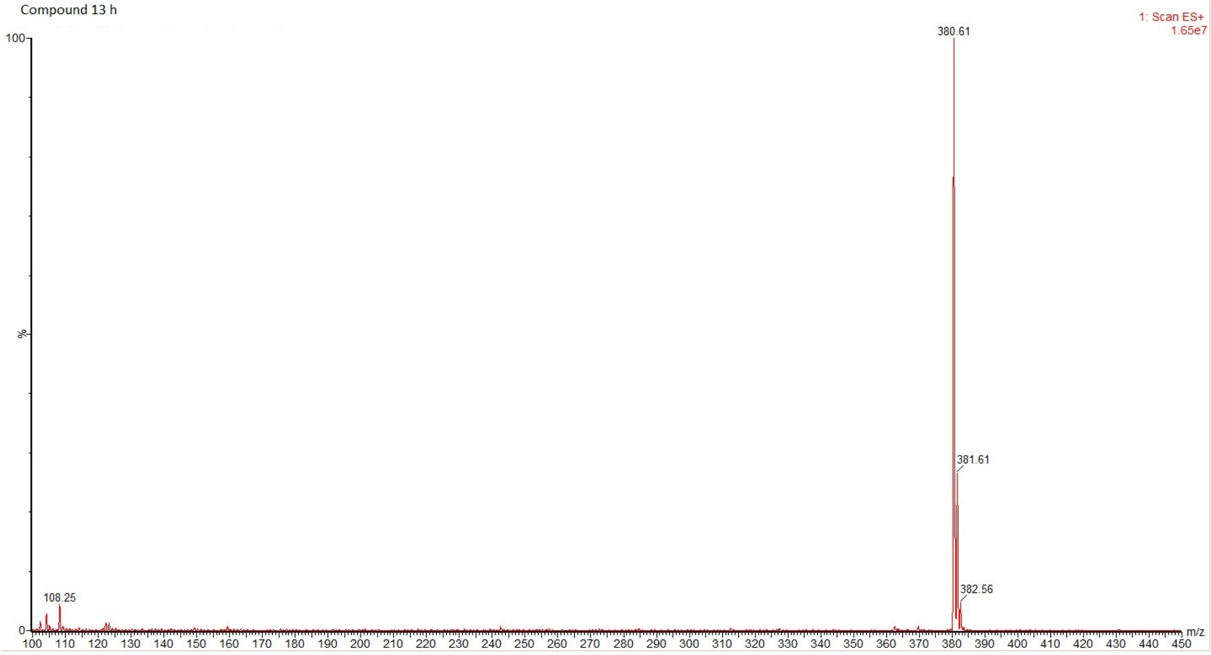

Supplement: Supplementary file 1 [file pharmaceuticals-15-00766-s001.zip › pharmaceuticals-1696796-supplementary.pdf]
